# Genome-wide scan highlights the role of candidate genes on phenotypic plasticity for age at first calving in Nellore heifers

**DOI:** 10.1038/s41598-020-63516-4

**Published:** 2020-04-15

**Authors:** Lucio F. M. Mota, Fernando B. Lopes, Gerardo A. Fernandes Júnior, Guilherme J. M. Rosa, Ana F. B. Magalhães, Roberto Carvalheiro, Lucia G. Albuquerque

**Affiliations:** 1 São Paulo State University (UNESP), School of Agricultural and Veterinarian Sciences, Via de Acesso Prof. Paulo Donato Castelane, Jaboticabal, SP 14884-900 Brazil; 2 Geneticist Cobb-Vantress, 305 E Main St, Siloam Springs, AR 72761 USA; 3 Department of Animal Sciences, University of Wisconsin-Madison, 1675 Observatory Dr., Madison, WI 53706 USA; 4 National Council for Science and Technological Development, Brasilia, DF 71605-001 Brazil

**Keywords:** Animal breeding, Robustness

## Abstract

Age at first calving (AFC) plays an important role in the economic efficiency of beef cattle production. This trait can be affected by a combination of genetic and environmental factors, leading to physiological changes in response to heifers’ adaptation to a wide range of environments. Genome-wide association studies through the reaction norm model were carried out to identify genomic regions associated with AFC in Nellore heifers, raised under different environmental conditions (EC). The SNP effects for AFC were estimated in three EC levels (Low, Medium, and High, corresponding to average contemporary group effects on yearling body weight equal to 159.40, 228.6 and 297.6 kg, respectively), which unraveled shared and unique genomic regions for AFC in Low, Medium, and High EC levels, that varied according to the genetic correlation between AFC in different EC levels. The significant genomic regions harbored key genes that might play an important biological role in controlling hormone signaling and metabolism. Shared genomic regions among EC levels were identified on BTA 2 and 14, harboring candidate genes associated with energy metabolism (*IGFBP2, IGFBP5, SHOX, SMARCAL1, LYN*, *RPS20*, *MOS*, *PLAG1*, *CHCD7*, and *SDR16C6*). Gene set enrichment analyses identified important biological functions related to growth, hormone levels affecting female fertility, physiological processes involved in female pregnancy, gamete generation, ovulation cycle, and age at puberty. The genomic regions highlighted differences in the physiological processes linked to AFC in different EC levels and metabolic processes that support complex interactions between the gonadotropic axes and sexual precocity in Nellore heifers.

## Introduction

In tropical beef cattle, animals are raised in a variety of production systems and exposed to a wide range of nutrition levels. These differences may cause genotype-environment (GxE) interaction on various productive traits^1–3^. GxE interaction occurs when the genetic variance and/or the classification of animals change according to the environment^4,5^. The re-ranking of animals caused by GxE interaction is an indication that some genomic regions might have different effects according to the environments^6,7^.

Integrating genotypic information with reaction norm (RN) models lead to the evaluation of GxE interaction more accurately compared to only pedigree-based analysis^3,8,9^. In addition, RN models can be combined with genome-wide association studies (GWAS) allowing to uncover genomic regions involved in animal adaptation by estimating SNP effects across environmental conditions^8,10^. In this context, a greater understanding can be obtained on how genetic variants are associated with reproductive traits and the environmental sensitivity by physiological adaptation.

The integration of GWAS with RN models has been shown to be a powerful approach to investigate the key regulators and genomic regions that explain the phenotypic variation across environments^10,11^. Moreover, pathway and gene network analyses from these results can be performed to uncover mechanisms whereby the environment can potentially affect the sexual precocity in cattle. Such knowledge regarding genomic regions and biological pathways involved with GxE interactions in Nellore heifers’ sexual precocity is important to identify molecular mechanisms underlying the phenotypic responses to different environments. Hence, this study was carried out to evaluate the changes in the SNP effect estimates, as well as the biological processes associated with age at first calving in three environmental conditions, combining RN models and GWAS.

## Materials and Methods

### Ethics approval

The animal procedures in this study were approved by Animal Care of the São Paulo State University (UNESP), School of Agricultural and Veterinary Science Ethical Committee (protocol number 18.340/16). All the data sampling was performed in accordance with CEUA/ FCAV-UNESP guidelines and regulations.

### Phenotypic and genotypic data

Age at first calving (AFC) records were obtained from 185,356 Nellore heifers belonging to three commercial breeding programs (DeltaGen, Paint – CRV Lagoa and Cia de Melhoramento), which are part of Alliance Nellore database (www.gensys.com.br). The animals, born between 1984 and 2014, were from 200 commercial herds widely distributed in the Midwest, Southeast, and Northeast of Brazil and show high connectedness by the common sires intensively used through artificial insemination (AI), with more than 50% of the calves born from AI.

The AFC was computed, in days, as the difference between the first calving date and heifer’s birth date. It was assumed that heifers were mated with sires with similar fertility and similar breeding value for gestation length because this information was not available for all sires. Previously to the beginning of each mating season, sires were assessed for fertility and those considered sub-fertile were discarded. Also, differences in breeding value for gestation length between sires are not greater than 10 days.

In general, heifers were exposed to two breeding seasons: an anticipated breeding season, occurring usually in the first trimester of each year, during which all heifers (irrespective of body weight and body condition score) are exposed to reproduction at about 16 months of age; and a regular breeding season, usually between November and January, in which cows are also exposed and the heifers have about 26 months of age. Heifers that did not conceive during the anticipated breeding season had another chance in the second breeding season and were discarded if they did not get pregnant. During the mating seasons, the heifers were either artificially inseminated or naturally mated (~50%). When a fixed time AI protocol was used, the entire contemporary group received the same protocol.

Contemporary groups (CG) for AFC were formed by concatenating heifers born in the same year and season, from the same farm and raised in the same management group at birth, weaning, and yearling. Observations outside the interval between 3.5 standard deviations below and above the mean of each CG and CG with less than 5 records were excluded. After quality control of phenotypic data, a total of 149,665 AFC records, distributed in 5,296 CG, with an average of 1052.78 ± 110.19 days, were used.

Genomic DNA of heifers was extracted from hair and of sires from semen. A total of 1,900 heifers and 1,129 sires were genotyped using the Illumina BovineHD BeadChip assay (770 k, Illumina Inc., San Diego, CA, USA) and 850 heifers using GeneSeek® Genomic Profiler HDi 75 K (GeneSeek In/c., Lincoln, NE). The heifers genotyped with the lower density panel (75 K) were imputed to the HD panel using FImpute v2.2^12^ with an expected imputation accuracy of 0.98, according to the imputation study performed by Carvalheiro *et al*.^13^. FImpute was run using pedigree information as well as parentage testing option. The detected parent-progeny conflicts (~3%) were corrected in the pedigree file. Quality control of genotypes was performed excluding non-autosomal regions. Autosomal markers presenting minor allele frequency (MAF) less than 0.03, significant deviation from Hardy–Weinberg equilibrium (P ≤ 10^−5^) and with call rate less than 0.95 were removed. Samples with a call rate lower than 0.95 were removed. After quality control, 2,650 heifers, 1,110 sires, and 446,554 SNPs remained in the dataset. Heifers of genotyped sires comprised 55.99% of phenotypic records (83,805 heifers), from those 1480 were genotyped. The genotyped sires were those more influential (higher number of progeny) for the studied herds, which were predominantly AI sires. The number of progenies by sire is shown in Supplementary Fig. S1.

### Statistical modeling

#### Environmental condition descriptor

The dataset used to evaluate the sensitivity of sexual precocity to environmental variation belonged to commercial herds with high diversity of management and different environmental conditions, since these commercial herds were located in regions with variations on annual precipitation from ~700 to ~3000 mm and exhibiting a dry season that may last up to 7 months. Remarkably, differences in nutritional levels by farms are common, for instance in some of the farms, the animals received protein and mineral supplementation, especially during the dry season, while in others only urea supplementation was offered. Details of the climate classification in the Midwest, Southeast, and Northeast of Brazil can been seen in Alvares *et al*.^14^.

The AFC genetic sensitivity to environmental changes was assessed through the reaction norm model^15,16^. In this context, the animal’s response to environmental condition changes was expressed as a function of a continuous environmental condition (EC) descriptor. In the lack of environmental condition descriptor information (e.g. temperature-humidity index, level of production, etc.), descriptors based on the CG solutions from phenotypic information, can be used. In this framework, the EC descriptor used to encompass the production level was based on the best linear unbiased estimates (BLUE) solutions of the contemporary group for yearling body weight (YBW), once it condenses the different management and environmental factors in which the animals were raised. We focused on YBW because the differences in production environments affecting YBW present an important effect on heifers’ early sexual puberty^2,3,17,18^.

The environmental descriptor used on RN model referred as environmental condition (EC), was the standardized BLUE of CG effects solutions for YBW obtained using an animal model considering the single-step GBLUP (ssGBLUP) method as follows:

$$y=X\beta +Za+e$$

where $y$ is the vector of YBW; $\beta$ is a vector with the fixed effects of CG (defined by animals born in the same year and season, and raised in the same farm and management group from birth to yearling) and age at recording as a linear co-variable; $a$ is a vector of additive genetic effects assumed normally distributed $N(0,H\otimes {\sigma }_{a}^{2})$, in which ${\sigma }_{a}^{2}$ is the additive genetic variance and $H$ is the pedigree-genomic relationship matrix ⊗ is the Kronecker product and $e$ is a residual vector assumed $N(0,I\otimes {\sigma }_{e}^{2})$, where $I$ is the identity matrix, and ${\sigma }_{e}^{2}$ is the residual variance. The $X$ and $Z$ are known as incidence matrices related to fixed and additive genetic effects, respectively.

The *H* is a matrix that combines pedigree and genomic information^19^ and its inverse (*H*^−1^) is given by: $${H}^{-1}={A}^{-1}+[\begin{array}{cc}0 & 0\\ 0 & {G}^{-1}-{A}_{22}^{-1}\end{array}]$$, where *A*^−1^ is the inverse of the pedigree relationship matrix, ${A}_{22}^{-1}$ represents the inverse of the pedigree relationship matrix for genotyped animals, and *G*^−1^ is the inverse of the genomic relationship matrix obtained according to VanRaden^20^.

The CG solutions ranged from 148.05 to 327.25 kg, with an average of 231.55 kg, highlighting the differences across the environmental conditions in which the heifers were raised. For more information about CG solutions see Supplementary Fig. S2. The EC descriptors were obtained by CG solutions that were standardized to show a mean value of 0 and standard deviation (sd) equal to 1, with values ranging from −3 to +3 sd. The Low (−3.0 sd), Medium (0.0 sd) and High (3.0 sd) values of EC levels corresponding to CG effects on YBW equal to 159.40, 228.6 and 297.6 kg, respectively. The number of animals and the phenotypic average for AFC across EC levels are in Supplementary Fig. S3.

### Reaction norm (RN) model

In order to estimate the genomic breeding value (GEBV) for AFC, a single-step genomic reaction norm model (ssGRN) was used as follows:

$${y}_{ij}={F}_{CG}+\mathop{\sum }\limits_{f=0}^{1}{\omega }_{f}{\varPhi }_{f}(E{C}_{j})+\mathop{\sum }\limits_{f=0}^{1}{\alpha }_{fi}{\varPhi }_{f}(E{C}_{j})+{e}_{ij}$$

where *y*_*ij*_ is the vector of AFC information of the animal *i* recorded in the level *j* of EC, *F*_*CG*_ is the fixed effect of CG, $${\omega }_{f}$$ are the *f-*th fixed regression coefficients (intercept and slope) on $${\varPhi }_{f}(E{C}_{j})$$; $${\varPhi }_{f}(E{C}_{j})$$ are the *f-*th Legendre polynomials corresponding to *EC* level *j* ($$E{C}_{j})$$, $${\alpha }_{fi}$$ are the random regression coefficients for additive effects of intercept and slope corresponding to animal *i* on EC level *j*, and $${e}_{ij}$$ is a random residual. Residual variances were considered to be heterogeneous across EC levels and five classes were determined using K-means clustering variance^21^. The residual class 1: EC level lower than −1.5; residual class 2: −1.5 ≤ EC level < −0.5; residual class 3: −0.5 ≤ EC level < 0.; residual class 4: 0.0 ≤ EC level < 1.5, and residual class 5: EC level higher than 1.5.

The ssGRN model was fitted considering the following assumptions for the random effects a = {*a*_*j*_} ∼ *N*(*0*, $$H\otimes [\begin{array}{c}{{\sigma }}_{a}^{2}\\ {{\sigma }}_{ab}\end{array}\begin{array}{c}{{\sigma }}_{ab}\\ {{\sigma }}_{a}^{2}\end{array}]$$): and e = {*e*_ij_}∼ *N*(*0*, *I ⊗ R*), where ${\sigma }_{a}^{2}$, ${\sigma }_{b}^{2}$ and ${\sigma }_{{\rm{ab}}}$ are variances for the intercept, slope and covariance between intercept and slope, respectively, and *R* is a diagonal residual variance matrix considering 5 heterogeneous classes. Estimates of (co)variance components were obtained by restricted maximum likelihood using the AIREMLF90 software^22^.

### Estimates of SNP effects and variance explained

Before GWAS analyses, animals with GEBV accuracies (based on prediction error variance) lower than 0.40 for reaction norm parameters (intercept and slope) were excluded. So, a total of 2,550 heifers and 1,023 sires were considered for estimating SNP effects for the slope and the intercept. Clustering analysis was performed to assess the possible occurrence of population substructure in the population, based on the genomic relationship matrix. Results did not indicate population stratification (Supplementary Fig. S4).

The SNP effects (${\hat{u}}_{kEC}$) were estimated across EC levels using the following equation: ${\hat{u}}_{kEC}={\alpha }_{k}\,\varPhi f{\prime}$, where ${\alpha }_{k}$ is the vector of intercept and slope estimates for the *k-th* SNP, and $\varPhi f{\prime}$ is the transposed vector of the *f-th* Legendre polynomials for each *EC* level. The SNP effects were obtained in three EC descriptors Low (−3.0 sd), Medium (0.0 sd) and High (3.0 sd). The genetic, phenotypic and residual variances, as well as heritability estimates for AFC across EC levels obtained with the RN model, are in Supplementary Fig. S5.

The percentage of genetic variance explained by the SNPs was estimated according to Wang *et al*.^23^: ${\sigma }_{{SNPEC}}^{2}=\frac{2{p}_{k}{q}_{k}{\hat{u}}_{kEC}}{{\sigma }_{a{\rm{EC}}}^{2}}\,\ast \,100$, where ${\sigma }_{{SNPEC}}^{2}$ is the percentage of variance explained by each SNP in the EC level (Low, Medium or High), ${\hat{u}}_{kEC}$ is a vector of the effect of the *kth* SNP in the EC level, ${p}_{k}$ and ${q}_{k}=(1-{p}_{k})$ are the allele frequencies, and ${\sigma }_{aEC}^{2}$ is the additive genetic variance for AFC in the EC level (Low, Medium or High).

### Statistical test for SNP marker effect

A statistical test on the SNP markers was performed by the standardization of the SNP effects^24^ as follows: ${z}_{kEC}={u}_{kEC}/\sqrt{{\sigma }_{{SNPEC}}^{2}}$, where ${z}_{kEC}$ is the z-score for SNP markers effects for each of three EC level. P-values for the SNP effects were computed as ${\rm{p}}-{\rm{value}}=2(1-\phi (|{z}_{k}|))$, where $\phi (|{z}_{k}|)$ is the cumulative function of the normal distribution for the z-score. The p-values were corrected for multiple tests using the false discovery rate (FDR)^25^: $fdr=\frac{{n}_{m}}{{n}_{msig}}\ast {p}_{{\rm{sig}}}$. The ${n}_{m}$ is the total number of SNP markers in the analysis (*n* = 446,554), ${n}_{msig}$ is the number of significant markers (p-value < 0.01) and ${p}_{{\rm{sig}}}$ represents the significance threshold (p-value < 0.01) used to consider a SNP as significant. The assessment of inflation/deflation factor were calculated as λ = median (p-value)/0.456, where values of *λ* between 1.0 and 1.1 were considered acceptable in GWAS^26^.

The linkage disequilibrium (LD) analysis was performed for the shared chromosome region between the three EC levels, by computing the r-square (*r*^2^)^27^ values for pairwise top SNP marker effect with markers around this region using the Gaston R package^28^.

### Gene mapping of significant SNP in each EC level

The SNP effects estimated for AFC in the three EC levels, Low (EC = −3.0), Medium (EC = 0.0) and High (EC = 3.0), were deemed significant when −*log*_10_ (*p-value*) > 6.0 (5% FDR). The Ensembl gene 94 database^29^ and BioMart R package^30^ were used to search genes located harboring 200 kb region of each significant SNP marker, using as reference the *Bos Taurus* UMD 3.1 assembly^31^. The Cattle QTL database (QTLdb)^32^ was used to identify previously detected quantitative trait loci (QTL) overlapping these regions, also considering the UMD v3.1 assembly sequence as the reference map. Biological mechanisms and pathways (Gene Ontology - GO) involving the candidate genes were identified using the clusterProfiler R Package^33^, separately for each EC level, considering as background the Bovine database^34^. The association of a given gene set with AFC in each EC level, with GO terms was assessed using a hypergeometric test^35^ considering a false discovery rate (FDR) of 5% for multiple test. The significance of GO terms was calculated as described by Boyle *et al*.^35^.

## Results and Discussion

### SNP effects in different environmental conditions

**Figure 1:**
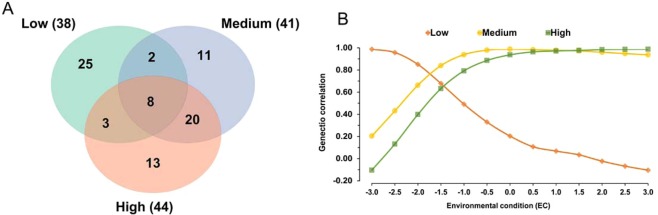
Venn diagram showing the numbers of overlapping significant SNP markers (*-log*_10_*(p-value)*> *6*) associated with age at first calving (AFC) in three environmental conditions (**A**) and genetic correlation estimates for AFC across Low, Medium and High EC levels (**B**).

The GWAS results for AFC showed a total of 38, 41 and 44 significant SNP markers (*-log*_10_*(p-value)* > *6*) for Low (BTA1, 2, 6, 14, 15, 17 and 27), Medium (BTA2, 3, 5, 14 and 18) and High (BTA2, 3, 5, 14 and 18) EC levels, respectively (Fig. 1A and Supplementary Table S1). Some of these SNP markers identified were shared among environmental conditions and can explain part of the genetic correlations for AFC among Low, Medium and High EC levels (Fig. 1B). Medium and High EC levels shared most of the significant SNP markers (20 SNPs). A total of 2 and 3 SNP markers were shared by Low x Medium and Low x High, respectively, and 8 markers were significant for all three EC levels (Fig. 1A).

The higher number of shared SNP markers for AFC between Medium and High EC levels could, in part, explain the higher genetic correlation coefficients estimated for AFC between these two EC levels (Fig. 1). When GxE interaction occurs, genotypes react differently according to the environmental levels^3,7^. Indeed, different genomic variants showed a specific effect on AFC in different environmental conditions (Fig. 1A). The genetic correlation for AFC in the three EC levels indicated an important environmental sensitivity resulting in a potential re-ranking of heifers under restrictive environmental conditions (Fig. 1B). These environmental differences are often associated with heat stress and seasonally poor nutrition, which causes a reduction in length of the estrous cycle, progesterone concentration and the developmental capacity of oocytes^36,37^.

**Figure 2:**
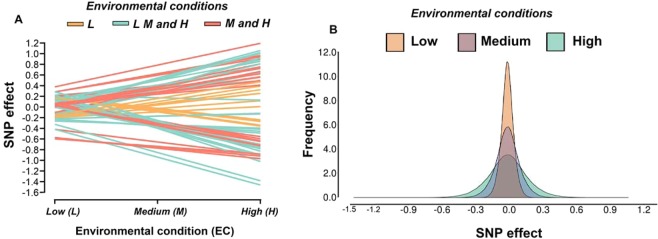
[**A**] Single nucleotide polymorphism (SNP) effect estimates significantly associated ($-{lo}{{g}}_{10}({p}-{value})\, > 6.0$) with age at first calving in Low (L), Medium (M) and High (H) environmental conditions (EC), the colors represents the shared or specific chromosome regions between EC levels and [**B**] SNP effect estimates distribution in three environmental conditions (Low, Medium and High).

The SNP markers detected (−*log*_10_ (*p-value*) > 6.0) for Nellore AFC showed re-ranking of their effects across EC levels, in which the effects in the Low condition were different from those in the High condition (Fig. 2A). The SNP effects changed in magnitude and direction of their effects according to the EC level (Fig. 2A). Some studies in dairy cattle^7,38^, pigs^8^ and beef cattle^3,10^ have shown that different environmental conditions can cause substantial changes in SNP effect estimates.

Higher percentages of the total genetic variance were explained by SNP markers in the High (18.13%) compared to the Low (6.63%) and the Medium (12.67%) environmental levels, as also observed for SNP marker effect dispersion (Fig. 2 A,B). The differences in the proportion of total genetic variance explained by SNP marker across the environmental levels indicated an important change of genetic variance effects according to EC levels. Hence, unraveling the importance of genomic regions through GWAS may contribute to design a more precise strategy for the selection of complex traits^38^ under different environmental conditions. Including genomic regions having environment-dependent sensitivity in statistical methods represents an additional biological insight to obtain more accurate genomic predictions and thus select Nellore heifers with greater genetic potential for sexual precocity and tolerance to harsh conditions. Streit *et al*.^11^, in dairy cattle, and Mota *et al*.^10^, in beef cattle, concluded that significant genomic regions across environments were environmental-dependent and associated with key physiological processes affecting the evaluated trait.

### Significant regions surround genes with EC level-specific effect

Inflation-factor ($\lambda$) estimates are 1.05, 1.08 and 1.09 for the Low, Medium and High EC levels (Supplementary Fig. S6), respectively and showed that the deviation of the observed test statistics from the theoretical quantiles was acceptable according to Georgiopoulos and Evangelou^39^.

**Figure 3:**
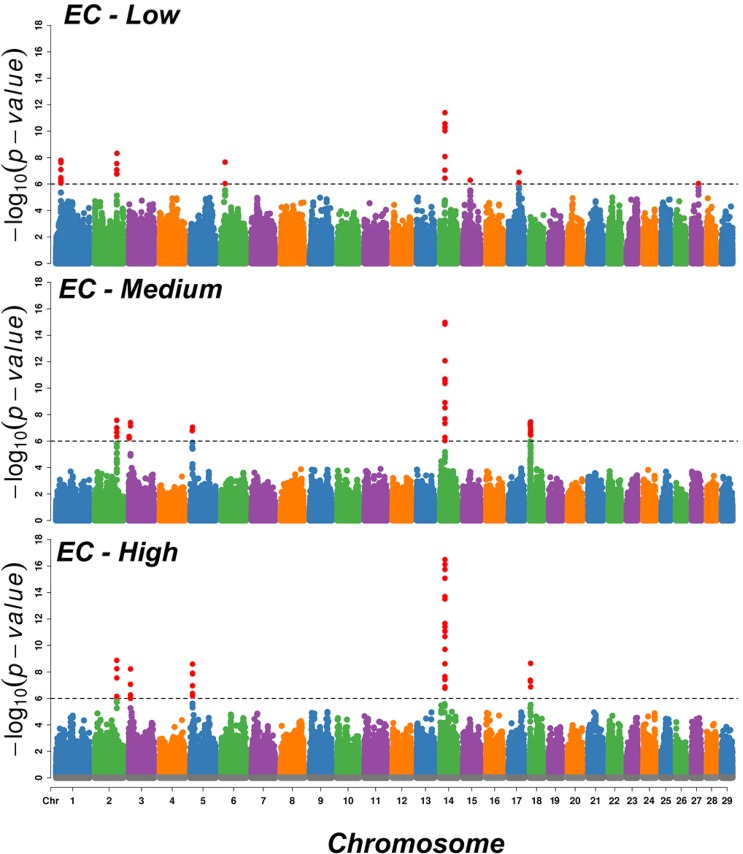
Manhattan plot of genome wide association in the three environmental conditions Low EC level = −3.0; Medium EC level = 0.0 and High EC level = 3.0 for age first calving (AFC) in Nellore heifers. The horizontal blackline represents the significance threshold *-log*_10_*(p-value)* > *6.0* for markers considering a FDR of 5%.

Specific genomic regions affecting AFC in each of the EC levels were identified as well as regions shared by two or three of the levels (Fig. 3). The specific regions for the Low EC level were mapped on BTA 1, 6, 15, 17 and 27, for the Medium EC level on BTA 3, 5 and 18 and for the High EC level on BTA14. The regions along the genome that were associated with AFC for each EC (Fig. 3), imply in different physiology mechanisms leading to sexual precocity in a specific environmental condition^40^.

**Figure 4:**
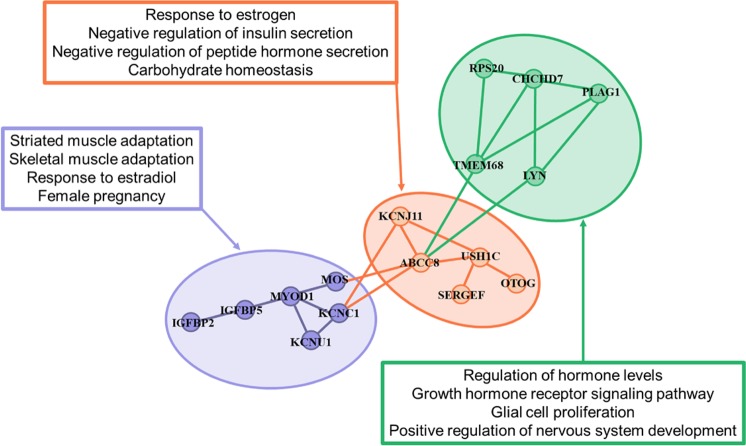
Functional gene ontology (GO) enrichment and gene networks among gene identified within 200 kb for SNP markers significantly affecting AFC in Low environmental condition (EC = −3.0). Color represents the major GO biological factors identified in the gene set enrichment (Supplementary Table S4).

The variants identified with a specific effect in the Low EC level overlapped the QTLs previously associated with body weight, tridecylic acid content, daughter pregnancy rate, and calving ease (Supplementary Table S2). A total of 9 genes were identified surrounding 200 kb from the position of each significant SNP identified on BTA 15 (35.34 Mb; *KCNC1, MYOD1* and *SERGEF*, and 35.64 Mb 35.64 Mb; *ABCC8, KCNJ11, NUCB2* and *USH1C*), BTA 6 (19.49 Mb *DKK2*) and BTA 27 (31.97 Mb *KCNU1)* for the Low EC level (Supplementary Table S3). These specific genes associated with Low EC level are involved in energetic metabolism by action on glucose homeostasis (GO:0042593, *KCNJ11* and *ABCC8*) and negative regulation of insulin secretion (GO:0046676, *KCNJ11* and *ABCC8*) (Fig. 4 and Supplementary Table S4). Such findings support the hypothesis that production systems on pasture conditions under Low EC level exhibit an important factor for delaying the precocity onset, through its impact on the concentration of circulating metabolites^18,36,41,42^.

Some candidate genes identified in Low EC level on BTA15 (*KCNJ1, KCNC1, MYOD1* and *SERGEF*) play a key role on response to estrogen (GO:0043627), regulation of estradiol (GO:0032355), hormone levels (GO:0010817), negative regulation of secretion (GO:0046888), muscle adaptation (GO:0014888 and GO:0043501) and are directly associated with growth rate, i.e. average daily gain (Fig. 4 and Supplementary Table S4). According to Rosales Nieto *et al*.^43^, sexual precocity is influenced by growth rate, which affects muscle and fat deposition. The *MYOD1* gene is a component of myogenic regulatory factors (*MYF-5, MYOD*, *myogenin* and *MRF4*) which is associated with muscle metabolism^36^. The myogenic factors are associated with endocrine factors, e.g. GH, estrogen, and IGF, with an important role in the regulation of muscle mass, fiber size, nutrient partitioning, and reproduction^44,45^. These factors might directly affect sexual precocity due to the effect of myogenic factors action on the insulin-glucose metabolic homeostasis with major effects on metabolic and endocrine roles on lower nutritional levels^36^.

The genes *ABCC8* (BTA15-35.64 Mb)*, DKK2* (BTA 6–19.49 Mb), and *NUCB2* (BTA15-35.64 Mb) are involved in energy and protein metabolism. The *NUCB2* gene is involved in hypothalamic pathways regulating feed intake and energy homeostasis, acting on leptin^46^. The *ABCC8* gene is associated with glucose homeostasis (GO:0042593), negative regulation of insulin secretion (GO:0046676) and negative regulation of peptide hormone secretion (GO:0090278) (Fig. 4 and Supplementary Table S4) with an important effect on regulating glucose metabolism by insulin secretion^47^. The *DKK2* gene increases adipogenesis and insulin resistance through on inhibition of the Wnt signaling pathway^48^. These metabolic responses to Low EC level involve the catabolic process, mainly to energy mobilization and protein degradation.

When animals are raised under Low environmental conditions, genes related to metabolism are required to recover the adaptability to poor nutrition conditions. The gene set identified in Low EC levels plays an important role to keep metabolic homeostasis, leading to different growth rates according to heifer adaptability allowing that precocious heifers reach their genetic potential. Ferraz Jr *et al*.^18^ observed that Nellore heifers selected to sexual precocity in the low nutritional level were able to attain the lowest age at puberty compared to heifers with high AFC, mainly by differences in *IGF1* and *Leptin* hormone level. In Low EC, physiological changes affecting levels of insulin and glucose could affect the AFC, because the oocyte quality and development of both oocyte and embryo respond directly to these metabolic inputs^40,49^.

**Figure 5:**
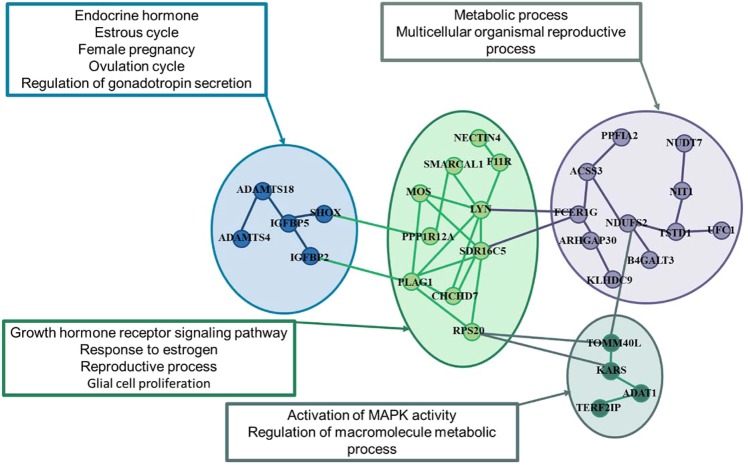
Functional gene ontology (GO) enrichment and gene networks among gene identified within 200 kb for SNP markers significantly affecting AFC in Medium environmental condition (EC = 0.0). Color represents the major GO biological factors identified in the gene set enrichment (Supplementary Table S5).

The SNP markers with a specific effect on AFC in Medium EC level harbor QTL regions associated with body weight, calving ease, calving interval, heifer pregnancy, and marbling score (Supplementary Table S2). A total of 4 genes (*ILDR2*, *POGK* and *TADA1*) were associated with these SNP markers on BTA 3 (2.11–2.12 Mb) and they are associated with important biological factors (Fig. 5 and Supplementary Table S5).

The *ILDR2* gene on BTA 3 (2.12 Mb) participate in a biological process that affects the circulating insulin and glucose levels and increases subcutaneous adipose tissue (Fig. 5 and Supplementary Table S5). The *ILDR2* gene is associated with lipid metabolism and plays a critical role in lipoprotein assembly, mainly on glucose and calcium homeostasis, in response to metabolic stress^50^.

The genes identified in Medium EC levels could reduce the AFC by their action in insulin, glucose levels, and energetic metabolism. It is likely that the improvement in EC levels affects heifers’ sexual precocity through changes in metabolic substrates (glucose and insulin) and their levels rather than a direct effect on reproductive hormones^17,37,49^. Samadi *et al*.^17^ observed that improved nutrition increases the blood concentration of insulin and glucose in Brahman heifers allowing lower AFC. The insulin and glucose provide signals in reproductive pathway regulations, affecting the oocytes quality and development, and the modulation of the gonadotropin-releasing hormone (*GnRH*) secretion^40^.

**Figure 6:**
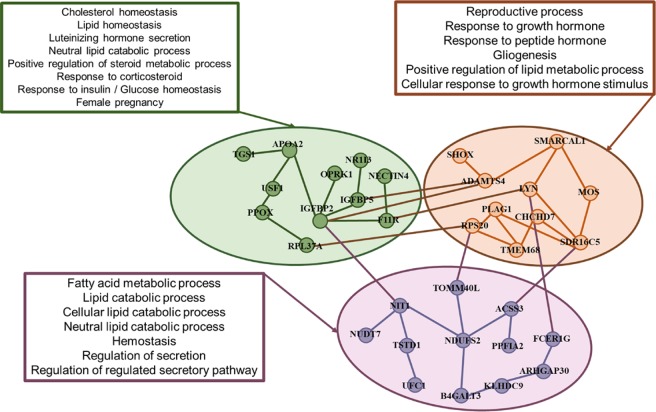
Functional gene ontology (GO) enrichment and gene networks among gene identified within 200 kb for SNP markers significantly affecting AFC in High environmental condition (EC = 3.0). Color represents the major GO biological factors identified in the gene set enrichment (Supplementary Table S6).

In High EC level, 14 SNP markers with specific effect for AFC were identified and surrounding two genes *OPRK1* (BTA14 23.39 Mb) and *TMEM68* (BTA 14 24.84 Mb). These genes are associated with neurons development related to the glial cell, a key component to the responses to growth factors and GnRH (gonadotropin-releasing hormone, GO:0032276 and GO:0032274) (Fig. 6 and Supplementary Table S6). It affects the integral functional elements of the synapses, responding to neuronal activity and regulating synaptic transmission and plasticity causing a reduction in the release of LH (GO:0032275) from the pituitary gland^51,52^ affected by *OPRK1* gene. The major impact of *OPRK1* in Nellore AFC is highlighted by the important biological process on negative regulation of LH secretion, which had been pointed out as a potential candidate in Brahman^53^ and Nellore^54^ cattle puberty. The *TMEM68* gene was identified to be associated with many reproductive traits in Nellore and Brahman^55^ and showed up-regulation in heifer blastocyst^56^, and is likely involved in energy metabolism and lipid turnover^57^. In addition, the sexual precocity is associated with physiological events linking major metabolic factors for the attainment of puberty^49^.

### Shared regions surround genes for AFC on Medium and High EC levels

Genomic regions on BTA 3, 5 and 18 associated with AFC (Supplementary Table S3) shared by Medium and High EC levels (Figs. 5 and 6) were associated to GO terms involved in regulation of endocrine process (GO:0044060); endocrine hormone secretion (GO:0051046, GO:1903305 and GO:0051046), estrogen levels (GO:0043627) energetic metabolism (GO:0016042, GO:0055088 and GO:0019216) and cellular response to insulin stimulus (GO:0032868 and GO:0043567) (Supplementary Tables S5 and 6).

The genes *ADAMTS4* on BTA3 (8.34 Mb) and *ADAMTS18* on BTA18 (4.63 Mb) affect folliculogenesis and ovulation, and they are also required for normal gonadal morphogenesis and function in cattle^58^. The genes *ADAMTS*4 and *ADAMTS*18 are associated with follicle growth because their expression are induced by the follicle-stimulating hormone (*FSH*), with a key role on tissue morphogenesis during embryonic development^58,59^. The *FCER1G* gene on BTA3 has been associated with QTLs related to pregnancy rate at first service, services per conception, and days open (Supplementary Table S2). This gene affects the immune response and presents an important effect on reproductive functions^60^.

The gene set (*ARHGAP30, USF1*) identified on BTA3 showed an important effect on the control of several genes related to lipid and glucose metabolism^61,62^. Hence, these genes can cause variation in AFC in Nellore heifers due to their influence on metabolic homeostasis, by direct action on the reproductive process, e.g. ovarian follicles, oocytes quality and embryos^18,49^. The genes *USP21* and *UFC1* are members of the Ubiquitin family gene and they were related to the metabolic process (GO:0008152), affecting the proteolysis regulating that represents a key aspect for the cellular function^63^.

The *DEDD* gene on BTA3 plays an important effect on the cell-cycle regulatory process, presenting roles in *SGK1* activity and *AKT* protein stability, with an essential effect on corporal glucose homeostasis^64^. The *DEDD* gene is indispensable for the support of female fertility in mice^64^ and it presents an effect on biological GO involved in female pregnancy associated with response to estrogen (GO:0060135) and reproductive process (GO:0022414). Glucose metabolism has shown an association with cattle reproduction^41,65^. In this context, the highest effect of genomic regions with a key role in glucose metabolism could lead to changes in reproductive endocrine hormones, e.g. *LH* and *FSH*^66^.

The SNP marker identified on BTA 5 (9.46–9.47 Mb), surrounding the PPP*1R12A* gene which plays a central role in a wide variety of biological processes. The *PPP1R12A* gene, also known as myosin phosphatase target subunit 1 (*MYPT1*), is involved in insulin signaling regulation by their action on insulin receptor substrate-1 (*IRS-1*)^67^. The effect of the *PPP1R12A* gene in sexual precocity could be related to metabolic homeostasis by action in insulin and glucose with an important role in the nervous system and ovary^68^.

The SNP markers found on BTA5 (10.85–10.88 Mb; Supplementary Table S3) are involved with energy and lipid metabolism by the action of the *ACSS3* gene that stimulates the utilization of acetate generating acetyl-CoA used in lipid synthesis or energy production^69^. The *PPFIA2* gene on BTA5 10.86 Mb members of *liprin* family show interaction with members of *LAR*, which have an important role in axon guidance and mammary gland development^70^. These genes are associated with the integrity of the central nervous system and represent an important role in the regulation of many aspects associated with the reproduction process^70^.

The gene *TERF2IP* on BTA 18 (3.02 Mb) is associated with various metabolic pathways, mainly regulating the NF-kB pathway^71^. The NF-kB pathway has a key role in the control of energy production by the regulation of glycolysis and cell respiration^72^.

The regions shared by Medium and High EC lead to differences in AFC through their action on energy metabolism. Energy metabolism is a pathway whereby higher EC levels lead to sexual precocity in Nellore cattle. The association of energy metabolism in sexual precocity occurs mainly because the glucose is the major energy source required to ovarian function and the luteinizing hormone (*LH*) secretion^49^. Brickell *et al*.^73^ observed that an increase of glucose levels in Holstein-Friesian heifers reduced their age at first breeding and calving. In Brahman heifers, the improvement of metabolic status increased glucose and lipid concentrations reducing the AFC^17^.

### Shared regions surround genes for AFC on low, medium and high EC levels

Results of the GWAS for AFC pointed out to shared genomic regions on BTA 2 (105.03–105.17 Mb) and BTA 14 (24.82–25.07 Mb) with the highest peak corresponding to marker rs137780934 located at 24.94 Mb on BTA14. The genomic regions surrounding the BovineHD0200030238 (rs443442023; BTA2 – 105.05 Mb) and BovineHD1400007242 (rs137780934; BTA14 – 24.94 Mb) markers have been pointed as a shared region that plays a key role in genetic differences in reproductive traits. These regions affect mostly growth and reproductive pathways, insulin growth factors (*IGF*) and hormonal levels^74–77^. Genomic regions on BTA14 (20–30 Mb) were identified by Fortes *et al*.^74^ harboring genes with shared effects on growth, carcass and reproductive traits, and showed a putative functional mutation. In the same region, Melo *et al*.^55^ identified genes with pleiotropic effect in reproductive traits in Nellore and Brahman cattle.

**Figure 7:**
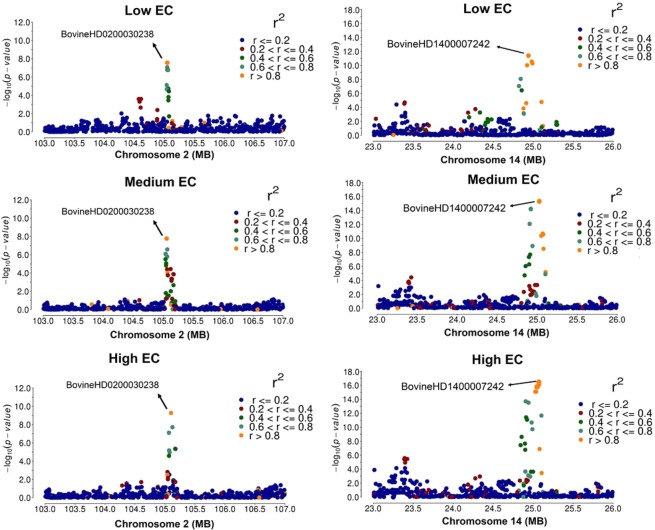
Regional association plot of BTA 2 (103.0–107.0 Mb) and 14 (23.0–26.0 Mb) for age at first calving (AFC) in three environmental conditions (Low, Medium and High). Linkage disequilibrium (LD) in r^2^ with SNP marker BovineHD0200030238 (rs443442023) on BTA2 and BovineHD1400007242 (rs137780934) on BTA14, represented according to the colors.

A linkage disequilibrium (LD) analyses on BTA 2 (103.0–107.0 Mb) and 14 (23.0–26.0 Mb) region was performed and indicated a strong association (from 0.60 to 0.80) surrounding the significant SNP marker BovineHD0200030238 (BTA2 105.039–105.17 Mb) and BovineHD1400007242 (BTA14 24.82–25.07 Mb) (Fig. 7). Previous QTLs studies detected genomic regions on BTA 2 and 14 play an important role in multiple traits (Supplementary Table S2)^74,78–81^. These genomic regions are known to act on age at puberty, calving to conception interval, the interval from first to last insemination, body weight and intramuscular fat (Supplementary Table S2).

**Figure 8:**
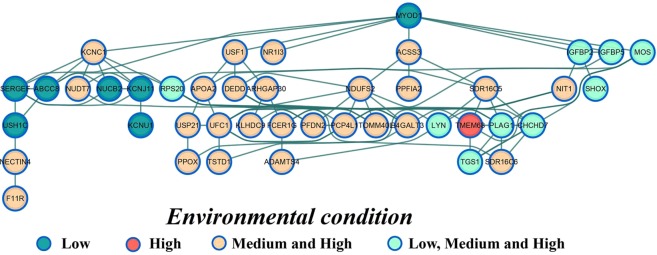
Network of candidate gene identified within 200 kb for SNP markers significantly affecting AFC in three environmental condition (Low, Medium and High). The gene network was built from known protein-protein interactions (edges) between gene products (nodes) using the string database for *Bos Taurus*. The node color represents the shared or specific genes across the Low, Medium and High environmental condition.

Shared genomic regions for AFC in three EC levels harbor candidate genes on BTA2 (*IGFBP2, IGFBP5, SHOX* and *SMARCAL1*) and BTA14 (*LYN*, *RPS20*, *MOS*, *PLAG1*, *CHCD7* and *SDR16C6*) with a striking effect in biological processes that might help explain the variability in the sexual precocity (Figs. 4–6). Taking into account the gene network analyses from GWAS across the three EC levels (Low, Medium and High) gave additional insights into the complex relationship among the specific and shared genes (Fig. 8). This result highlights the key role for molecular pleiotropy associated with the shared and specific genes across the EC level, which are likely to play an important role in the genetic architecture for sexual precocity in heifers raised under harsh conditions (Fig. 8).

These genomic regions on BTA 2 and 14 have been associated with hormone secretion (GO:0048545, GO:0060416, GO:0060986 and GO:0010817) and regulation of gonadotropin release (follicle-stimulating hormone - *FSH* and luteinizing hormone - *LH*). They are associated also with negative regulation of gonadotropin secretion (GO:0032276) and ovulation cycle (GO:0042698), affecting the estrous cycle by changes in ovarian follicle growth. Additionally, both regions affect endocrine hormone secretion (GO:0060986) with a key role in regulatory pathways of sexual precocity leading to a metabolic resilience^78^. The metabolic resilience to environmental conditions could explain their relationship with sexual precocity affecting factors related to growth rate and body condition.

Genes mapped on BTA14, particularly the gene set (*PLAG1, CHCHD7, LYN, MOS, PENK* and *RPS20*), highlight the effect on responses to the endogenous stimulus (GO:0009719; response to the hormone, response to fibroblast growth factor and response to transforming growth factor-beta). Furthermore, they have been associated with growth hormone (GO:0060416; growth hormone receptor signaling pathway) and response to hormone stimulus, resulting in a change of cellular activity and insulin levels (GO:0032868; insulin receptor signaling pathway). Such regions were also related to IGF1 (insulin-like growth factor 1) hormone levels due to polymorphisms on BTA14^82^ that have been significantly associated with the reduction in *IGF1* levels, fat depth and beginning of puberty in females and males^80–83^. Indeed, a link between the growth and reproductive traits in beef heifers has been previously reported by a response to these biological factors found in this study^17,18,42,49,74^.

The genomic region on BTA14 (BovineHD1400007242; rs137780934) has a key role on the puberty onset through GH signaling^84^. Hence, the *PLAG1* gene can affect the beginning of puberty in heifers through GH signaling and its direct effect on *IGF1* levels^85^. In addition, the *PLAG1* region was associated with delayed puberty by the increased growth, which affects body structure and growth rate, leading to delays in reproductive precocity in heifers until an adequate body condition to body size relation is achieved^86^.

The genomic regions on BTA 2 harbor the gene set (*IGFBP2, IGFBP5, SHOX* and *SMARCAL1*). The genes *IGFBP2* and *IGFBP5* have an important role in glucose metabolism of cattle, by the regulation of the bioactivity of *IGF1* and *IGF2* in the ovary^41^. In this sense, the *IGFBP* has been pointed out as a key factor to control follicle growth by sensitivity to gonadotropins^87^. Perhaps, the key function of *IGFBP2* and *IGFBP5* are tied to AFC across EC levels through a hormonal link that involves *GH*, *IGF1*, glucose and insulin. These metabolic signals affect the sexual precocity through direct effects on ovarian cells as well as on gonadotropins secretion^66^.

The gene set on BTA 2 (*IGFBP2* and *IGFBP5*) and on BTA 14 (*PLAG1, CHCHD7* and *LYN*) could have their action linked by physiological mechanisms affecting the reproductive traits by their effects on major pathways, such as insulin, glucose and *IGF* systems (Figs. 4–6). They are an important signal that allows associating the reproductive events to nutritional conditions. These factors are associated with the regulation of reproductive hormones and interact with ovarian activity, embryo development, oocyte production, and quality in cattle^77,88–90^.

The GWAS results for AFC in different environmental conditions showed strong evidence of genomic regions affecting physiological events, with changes on circulating metabolic signals, which result in the activation of the hypothalamus-pituitary-adrenal (HPA) axis. Thus, it is possible that differences in EC levels affect physiological response on AFC. According to Rhimd^91^ and Evans *et al*.^92^, harsh conditions and climate changes have an important effect on neuroendocrine mechanisms, changing the patterns of reproductive efficiency affecting puberty, estrus, and ovulation.

## Conclusion

Combining genome-wide scan and reaction norm models helped to identify genomic regions associated with age at first calving (AFC) in Nellore heifers in different environmental conditions. These genomic regions showed strong environmental dependence. Genomic regions identified in Low EC level BTA 1, 6, 15, 17 and 27 confirmed the importance of metabolic homeostasis on sexual precocity in restricted environments. In Medium EC levels the genomic regions on BTA 3 and 18 highlighted the role of energy and lipid metabolism involved in the reproductive pathway. In the High EC level, the genomic region on BTA14 acts on metabolic substrates. The significant genomic regions suggest that common variation in AFC trait on Medium and High EC is essential by controlling a key role in the regulation of physiological mechanisms of reproductive precocity. The shared genomic regions on BTA 2 and 14 in Low, Medium and High EC levels are directly implicated in the regulation of reproductive pathways underlying endocrine parameters associated with precocity. Overall, the major genomic regions uncovered across EC levels shared or with a specific effect are related to major modulators for metabolic adaptations to different environmental conditions allowing the heifers’ sexual precocity.

## Supplementary information

Supplementary Information.

## Data Availability

The Nellore phenotypic and genotypic information are not publicly available because they belong to commercial breeding programs. The data are available for academic use from the authors upon reasonable request.

## References

[1] Cardoso FF, Tempelman RJ (2012). Linear reaction norm models for genetic merit prediction of Angus cattle under genotype by environment interaction. J. Anim. Sci..

[2] Chiaia HLJ (2015). Genotype × environment interaction for age at first calving, scrotal circumference, and yearling weight in Nellore cattle using reaction norms in multitrait random regression models. J. Anim. Sci..

[3] Mota LFM (2020). Genomic reaction norm models exploiting genotype × environment interaction on sexual precocity indicator traits in Nellore cattle. Anim. Genet..

[4] Calus MPL, Veerkamp RF (2003). Estimation of environmental sensitivity of genetic merit for milk production traits using a random regression model. J. Dairy Sci..

[5] Calus MPL, Bijma P, Veerkamp RF (2004). Effects of data structure on the estimation of covariance functions to describe genotype by environment interactions in a reaction norm model. Genet. Sel. Evol..

[6] Hayes BJ, Daetwyler HD, Goddard ME (2016). Models for Genome × Environment Interaction: Examples in Livestock. Crop Sci..

[7] Lillehammer M (2008). Quantitative Trait Locus-by-Environment Interaction for Milk Yield Traits on Bos taurus Autosome 6. Genetics.

[8] Silva FF (2014). Sire evaluation for total number born in pigs using a genomic reaction norms approach. J. Anim. Sci..

[9] Zhang Z (2019). Genotype-by-environment interaction of fertility traits in Danish Holstein cattle using a single-step genomic reaction norm model. Heredity (Edinb)..

[10] Mota RR (2017). Analyses of reaction norms reveal new chromosome regions associated with tick resistance in cattle. Animal.

[11] Streit M (2013). Using genome-wide association analysis to characterize environmental sensitivity of milk traits in dairy cattle. G3 Genes|Genomes|Genetics.

[12] Sargolzaei M, Chesnais JP, Schenkel FS (2014). A new approach for efficient genotype imputation using information from relatives. BMC Genomics.

[13] Carvalheiro R (2014). Accuracy of genotype imputation in Nelore cattle. Genet. Sel. Evol..

[14] Alvares CA, Stape JL, Sentelhas PC, de Moraes Gonçalves JL, Sparovek G (2013). Köppen’s climate classification map for Brazil. Meteorol. Zeitschrift.

[15] Falconer DS (1990). Selection in different environments: effects on environmental sensitivity (reaction norm) and on mean performance. Genet. Res..

[16] Robertson A (1959). The Sampling Variance of the Genetic Correlation Coefficient. Biometrics.

[17] Samadi F, Blache D, Martin GB, D’Occhio MJ (2014). Nutrition, metabolic profiles and puberty in Brahman (Bos indicus) beef heifers. Anim. Reprod. Sci..

[18] Ferraz MVC (2018). A combination of nutrition and genetics is able to reduce age at puberty in Nelore heifers to below 18 months. animal.

[19] Aguilar I (2010). A unified approach to utilize phenotypic, full pedigree, and genomic information for genetic evaluation of Holstein final score. J. Dairy Sci..

[20] VanRaden PM (2008). Efficient methods to compute genomic predictions. J. Dairy Sci..

[21] Hartigan JA, Wong MA (1979). A k-means clustering algorithm. Appl. Stat..

[22] Misztal, I. *et al*. Manual for BLUPF90 family of programs. Univ. Georg. Athens, USA (2015).

[23] Wang H, Misztal I, Aguilar I, Legarra A, Muir WM (2012). Genome-wide association mapping including phenotypes from relatives without genotypes. Genet. Res. (Camb)..

[24] Gualdrón Duarte JL (2014). Rapid screening for phenotype-genotype associations by linear transformations of genomic evaluations. BMC Bioinformatics.

[25] Qu H-Q, Tien M, Polychronakos C (2010). Statistical significance in genetic association studies. Clin. Investig. Med..

[26] Devlin B, Roeder K (1999). Genomic control for association studies. Biometrics.

[27] Hill WG, Robertson A (1968). Linkage disequilibrium in finite populations. Theor. Appl. Genet..

[28] Perdry H, Dandine-Roulland L (2018). gaston — Genetic Data Handling (QC, GRM, LD, PCA) & Linear Mixed Models. R Packag. version 1.5.5.

[29] Aken BL (2016). The Ensembl gene annotation system. Database J. Biol. databases curation.

[30] Durinck S, Spellman PT, Birney E, Huber W (2009). Mapping identifiers for the integration of genomic datasets with the R/Bioconductor package biomaRt. Nat. Protoc..

[31] Zimin AV (2009). A whole-genome assembly of the domestic cow, Bos taurus. Genome Biol..

[32] Hu Z-L, Park CA, Reecy JM (2016). Developmental progress and current status of the Animal QTLdb. Nucleic Acids Res..

[33] Yu G, Wang L-G, Han Y, He Q-Y (2012). clusterProfiler: an R Package for Comparing Biological Themes Among Gene Clusters. OMICS.

[34] Carlson, M. org.Bt.eg.db: Genome wide annotation for Bovine. R Packag. version 3.8.2., 10.18129/B9.bioc.org.Bt.eg.db (2020).

[35] Boyle EI (2004). GO::TermFinder-open source software for accessing Gene Ontology information and finding significantly enriched Gene Ontology terms associated with a list of genes INTRODUCTION: MOTIVATION AND DESIGN. Bioinforma. Appl. NOTE.

[36] Chagas LM (2007). Invited review: new perspectives on the roles of nutrition and metabolic priorities in the subfertility of high-producing dairy cows. J. Dairy Sci..

[37] Ashworth CJ, Toma LM, Hunter MG (2009). Nutritional effects on oocyte and embryo development in mammals: implications for reproductive efficiency and environmental sustainability. Philos. Trans. R. Soc. B Biol. Sci..

[38] Lillehammer M, Hayes BJ, Meuwissen THE, Goddard ME (2009). Gene by environment interactions for production traits in Australian dairy cattle. J. Dairy Sci..

[39] Georgiopoulos G, Evangelou E (2016). Power considerations for λ inflation factor in meta-analyses of genome-wide association studies. Genet. Res. (Camb)..

[40] Garnsworthy PC, Sinclair KD, Webb R (2008). Integration of physiological mechanisms that influence fertility in dairy cows. animal.

[41] Lucy MC, Butler ST, Garverick HA (2014). Endocrine and metabolic mechanisms linking postpartum glucose with early embryonic and foetal development in dairy cows. animal.

[42] Samadi F, Phillips NJ, Blache D, Martin GB, D’Occhio MJ (2013). Interrelationships of nutrition, metabolic hormones and resumption of ovulation in multiparous suckled beef cows on subtropical pastures. Anim. Reprod. Sci..

[43] Rosales Nieto CA (2013). Selection for superior growth advances the onset of puberty and increases reproductive performance in ewe lambs. Animal.

[44] Brameld JM, Daniel ZCTR (2008). In utero effects on livestock muscle development and body composition. Aust. J. Exp. Agric..

[45] Yu M (2015). Insulin-like growth factor-1 (IGF-1) promotes myoblast proliferation and skeletal muscle growth of embryonic chickens via the PI3K/Akt signalling pathway. Cell Biol. Int..

[46] Lents CA (2013). Effects of nesfatin-1 on food intake and LH secretion in prepubertal gilts and genomic association of the porcine NUCB2 gene with growth traits. Domest. Anim. Endocrinol..

[47] Chandran S, Yap F, Hussain K, Kong L (2014). Molecular mechanisms of protein induced hyperinsulinaemic hypoglycaemia. World J Diabetes.

[48] Christodoulides C, Lagathu C, Sethi JK, Vidal-Puig A (2009). Adipogenesis and WNT signalling. Trends Endocrinol. Metab..

[49] D’Occhio MJ, Baruselli PS, Campanile G (2019). Influence of nutrition, body condition, and metabolic status on reproduction in female beef cattle: A review. Theriogenology.

[50] Watanabe K (2013). ILDR2: An Endoplasmic Reticulum Resident Molecule Mediating Hepatic Lipid Homeostasis. PLoS One.

[51] Yamamuro K, Kimoto S, Rosen KM, Kishimoto T, Makinodan M (2015). Potential primary roles of glial cells in the mechanisms of psychiatric disorders. Front. Cell. Neurosci..

[52] Perea G, Araque A (2005). Glial calcium signaling and neuron–glia communication. Cell Calcium.

[53] Fortes MRS (2012). Finding genes for economically important traits: Brahman cattle puberty. Anim. Prod. Sci..

[54] Mota RR (2017). Genome-wide association study and annotating candidate gene networks affecting age at first calving in Nellore cattle. J. Anim. Breed. Genet..

[55] Melo TP (2018). Multitrait meta-analysis identified genomic regions associated with sexual precocity in tropical beef cattle. J. Anim. Sci..

[56] Carter F (2010). Effect of elevated circulating progesterone concentration on bovine blastocyst development and global transcriptome following endoscopic transfer of *in vitro* produced embryos to the bovine oviduct. Biol. Reprod..

[57] Chang P (2017). Molecular identification of transmembrane protein 68 as an endoplasmic reticulum-anchored and brain-specific protein. PLoS One.

[58] Madan P (2003). Expression of messenger RNA for ADAMTS subtypes changes in the periovulatory follicle after the gonadotropin surge and during luteal development and regression in cattle. Biol. Reprod..

[59] Sayasith K, Lussier J, Sirois J (2013). Molecular characterization and transcriptional regulation of a disintegrin and metalloproteinase with thrombospondin motif 1 (ADAMTS1) in bovine preovulatory follicles. Endocrinology.

[60] Ortega MS (2017). Association of single nucleotide polymorphisms in candidate genes previously related to genetic variation in fertility with phenotypic measurements of reproductive function in Holstein cows. J. Dairy Sci..

[61] Wu S (2010). Upstream transcription factor 1 influences plasma lipid and metabolic traits in mice. Hum. Mol. Genet..

[62] Haas BE (2012). Adipose Co-expression networks across Finns and Mexicans identify novel triglyceride-associated genes. BMC Med. Genomics.

[63] Ernst A (2013). A strategy for modulation of enzymes in the ubiquitin system. Science.

[64] Mori M (2011). Death effector domain–containing protein (DEDD) is required for uterine decidualization during early pregnancy in mice. J. Clin. Invest..

[65] Sutton-McDowall ML, Gilchrist RB, Thompson JG (2010). The pivotal role of glucose metabolism in determining oocyte developmental competence. Reproduction.

[66] Lucy MC (2008). Functional differences in the growth hormone and insulin-like growth factor axis in cattle and pigs: implications for post-partum nutrition and reproduction. Reprod. Domest. Anim..

[67] Pham K (2012). Insulin-stimulated phosphorylation of protein phosphatase 1 regulatory subunit 12B revealed by HPLC-ESI-MS/MS. Proteome Sci..

[68] Neganova I (2007). Role of central nervous system and ovarian insulin receptor substrate 2 signaling in female reproductive function in the mouse. Biol. Reprod..

[69] Valour D (2014). Energy and lipid metabolism gene expression of D18 embryos in dairy cows is related to dam physiological status. Physiol. Genomics.

[70] Schaapveld RQJ (1997). Impaired mammary gland development and function in mice lacking LAR receptor-like tyrosine phosphatase activity. Dev. Biol..

[71] Yeung F (2013). Nontelomeric role for Rap1 in regulating metabolism and protecting against obesity. Cell Rep..

[72] Tornatore L, Thotakura AK, Bennett J, Moretti M, Franzoso G (2012). The nuclear factor kappa B signaling pathway: integrating metabolism with inflammation. Trends Cell Biol..

[73] Brickell JS, Bourne N, McGowan MM, Wathes DC (2009). Effect of growth and development during the rearing period on the subsequent fertility of nulliparous Holstein-Friesian heifers. Theriogenology.

[74] Fortes MRS (2013). Evidence for pleiotropism and recent selection in the PLAG1 region in Australian Beef cattle. Anim. Genet..

[75] Takasuga A (2016). PLAG1 and NCAPG-LCORL in livestock. Anim. Sci. J..

[76] Karim L (2011). Variants modulating the expression of a chromosome domain encompassing PLAG1 influence bovine stature. Nat. Genet..

[77] Littlejohn M (2012). Genetic variation in PLAG1 associates with early life body weight and peripubertal weight and growth in Bos taurus. Anim. Genet..

[78] Khatkar MS, Randhawa IAS, Raadsma HW (2014). Meta-assembly of genomic regions and variants associated with female reproductive efficiency in cattle. Livest. Sci..

[79] G T Pereira A (2016). Pleiotropic genes affecting carcass traits in Bos indicus (Nellore) cattle are modulators of growth. PLoS One.

[80] Fortes MRS (2013). Genomic regions associated with fertility traits in male and female cattle: advances from microsatellites to high-density chips and beyond. Anim. Reprod. Sci..

[81] Fortes MRS (2016). Polymorphisms and genes associated with puberty in heifers. Theriogenology.

[82] Fortes MRS, Reverter A, Kelly M, McCulloch R, Lehnert SA (2013). Genome-wide association study for inhibin, luteinizing hormone, insulin-like growth factor 1, testicular size and semen traits in bovine species. Andrology.

[83] Hawken RJ (2012). Genome-wide association studies of female reproduction in tropically adapted beef cattle. J. Anim. Sci..

[84] Pinilla L, Aguilar E, Dieguez C, Millar RP, Tena-Sempere M (2012). Kisspeptins and reproduction: physiological roles and regulatory mechanisms. Physiol. Rev..

[85] Fortes MRS, Reverter A, Hawken RJ, Bolormaa S, Lehnert SA (2012). Candidate genes associated with testicular development, sperm quality, and hormone levels of inhibin, luteinizing hormone, and insulin-like growth factor 1 in Brahman bulls. Biol. Reprod..

[86] Velazquez MA (2005). The usefulness of a single measurement of insulin-like growth factor-1 as a predictor of embryo yield and pregnancy rates in a bovine MOET program. Theriogenology.

[87] Llewellyn S (2007). Effect of negative energy balance on the insulin-like growth factor system in pre-recruitment ovarian follicles of post partum dairy cows. Reproduction.

[88] Robinson RS, Mann GE, Gadd TS, Lamming GE, Wathes DC (2000). The expression of the IGF system in the bovine uterus throughout the oestrous cycle and early pregnancy. J. Endocrinol..

[89] Velazquez MA, Zaraza J, Oropeza A, Webb R, Niemann H (2009). The role of IGF1 in the *in vivo* production of bovine embryos from superovulated donors. Reproduction.

[90] Fenwick MA (2008). Negative energy balance in dairy cows is associated with specific changes in IGF-binding protein expression in the oviduct. Reproduction.

[91] Rhind SM (2004). Effects of maternal nutrition on fetal and neonatal reproductive development and function. Anim. Reprod. Sci..

[92] Evans NP, Bellingham M, Robinson JE (2016). Prenatal programming of neuroendocrine reproductive function. Theriogenology.

