# Supplementary material for: Genome-wide scan highlights the role of candidate genes on phenotypic plasticity for age at first calving in Nellore heifers
Source: Sci Rep. 2020 Apr 15;10:6481. doi: 10.1038/s41598-020-63516-4 (PMC7160148; doi:10.1038/s41598-020-63516-4)
Supplement: Supplementary file 1 — Supplementary Information. [file 41598_2020_63516_MOESM1_ESM.docx]

# Genome-wide scan highlights the role of candidate genes on phenotypic plasticity for age at first calving in Nellore heifers

Lucio F.M. Mota^1*^, Fernando B. Lopes^2^, Gerardo A. Fernandes Júnior^1^, Guilherme J. M. Rosa^3^, Ana F.B. Magalhães^1^, Roberto Carvalheiro^1,4^, Lucia G. Albuquerque^1,4*^

^1^São Paulo State University (UNESP), School of Agricultural and Veterinarian Sciences, Via de Acesso Prof. Paulo Donato Castelane, Jaboticabal - SP, 14884-900, Brazil.

^2^ Geneticist Cobb-Vantress, 305 E Main St, Siloam Springs - AR 72761, USA.

^3^Department of Animal Sciences, University of Wisconsin-Madison, 1675 Observatory Dr., Madison - WI, 53706, USA.

^4^National Council for Science and Technological Development, Brasilia - DF, 71605-001, Brazil

* Correspondence and requests for materials should be addressed to Lucio F.M Mota (flaviommota.zoo@gmail.com) and Lucia G. Albuquerque (galvao.albuquerque@unesp.br)

# Supplementary information


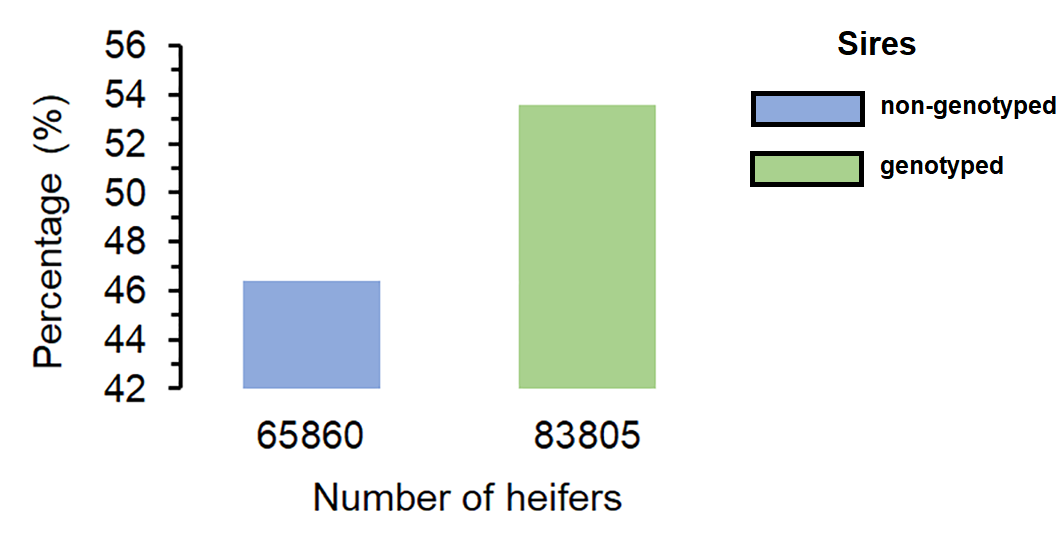


Supplementary Figure S1. Number of heifers (x axis) by non-genotyped (44.01 %) and genotyped (55.99 %) sires and frequency of the phenotypic information for age at first calving (y axis).


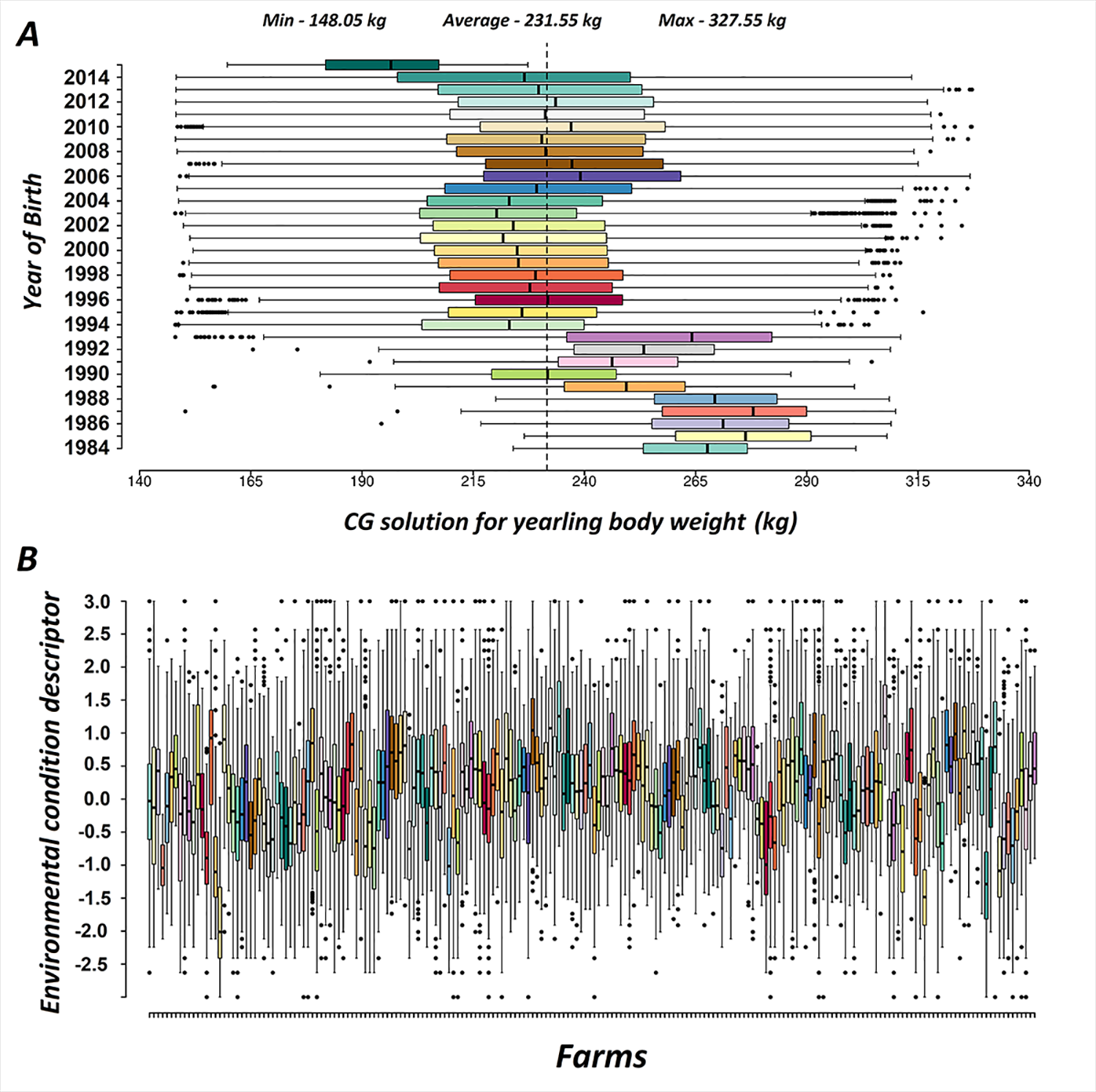


Supplementary Figure S2 – (A) Boxplot of contemporary group (CG) solutions (best linear unbiased estimates - BLUE) for yearling body weight (kg) of Nellore heifers by year (from 1984 to 2015). The dashed line represents the average for CG solutions (231.55 kg). (B) Boxplot of environmental conditions descriptor (EC) as standardized BLUE of CG effect solutions for yearling body weight across the farms.

Supplementary Figure S3. Number of records across environmental condition descriptors (bars, left axis) and phenotypic average for age at first calving (dot, right axis) in Nellore heifers.


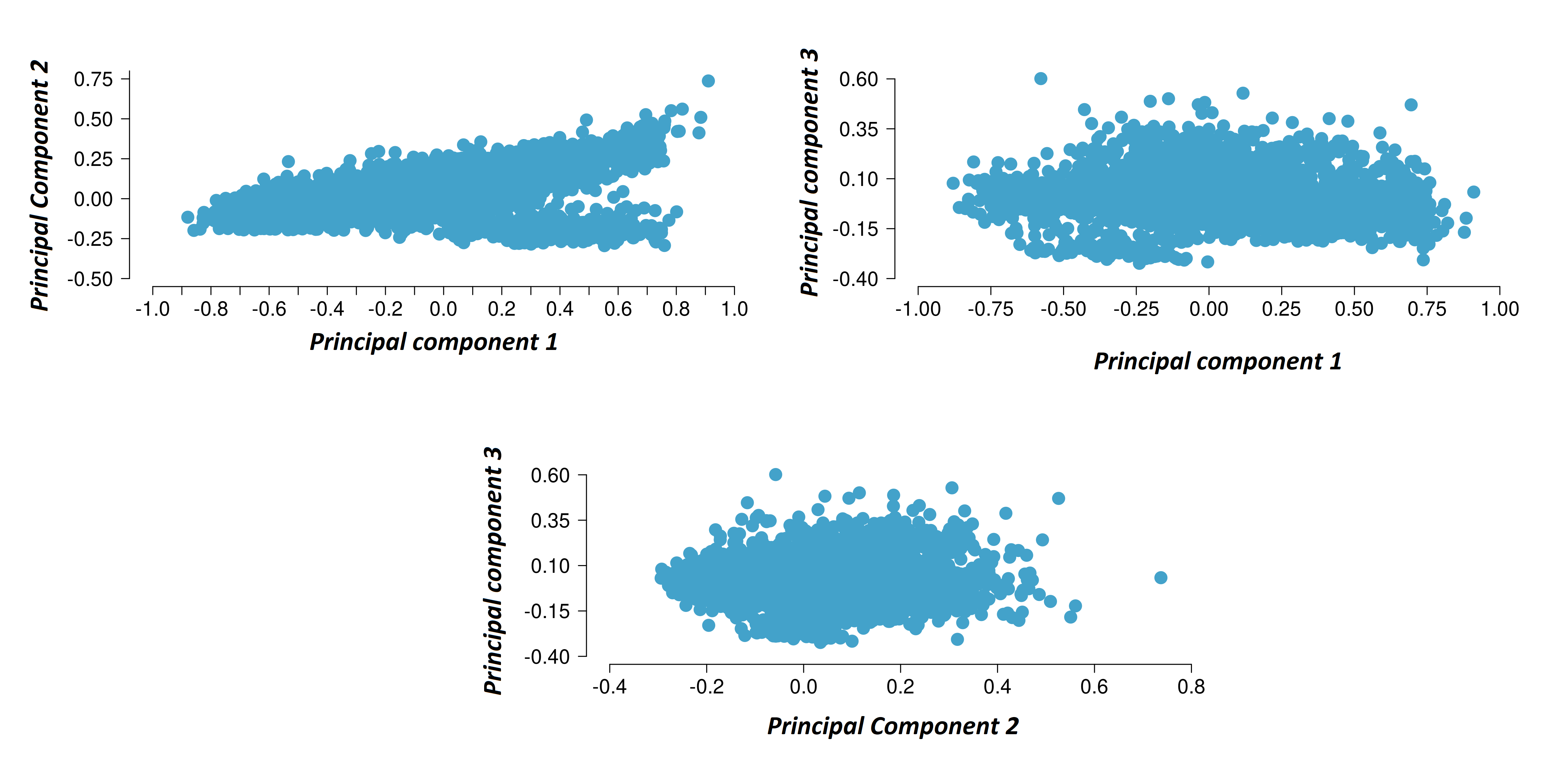


Supplementary Figure S4 – Plot of the three first principal components analysis based on the genomic kinship coefficient in Nellore cattle.


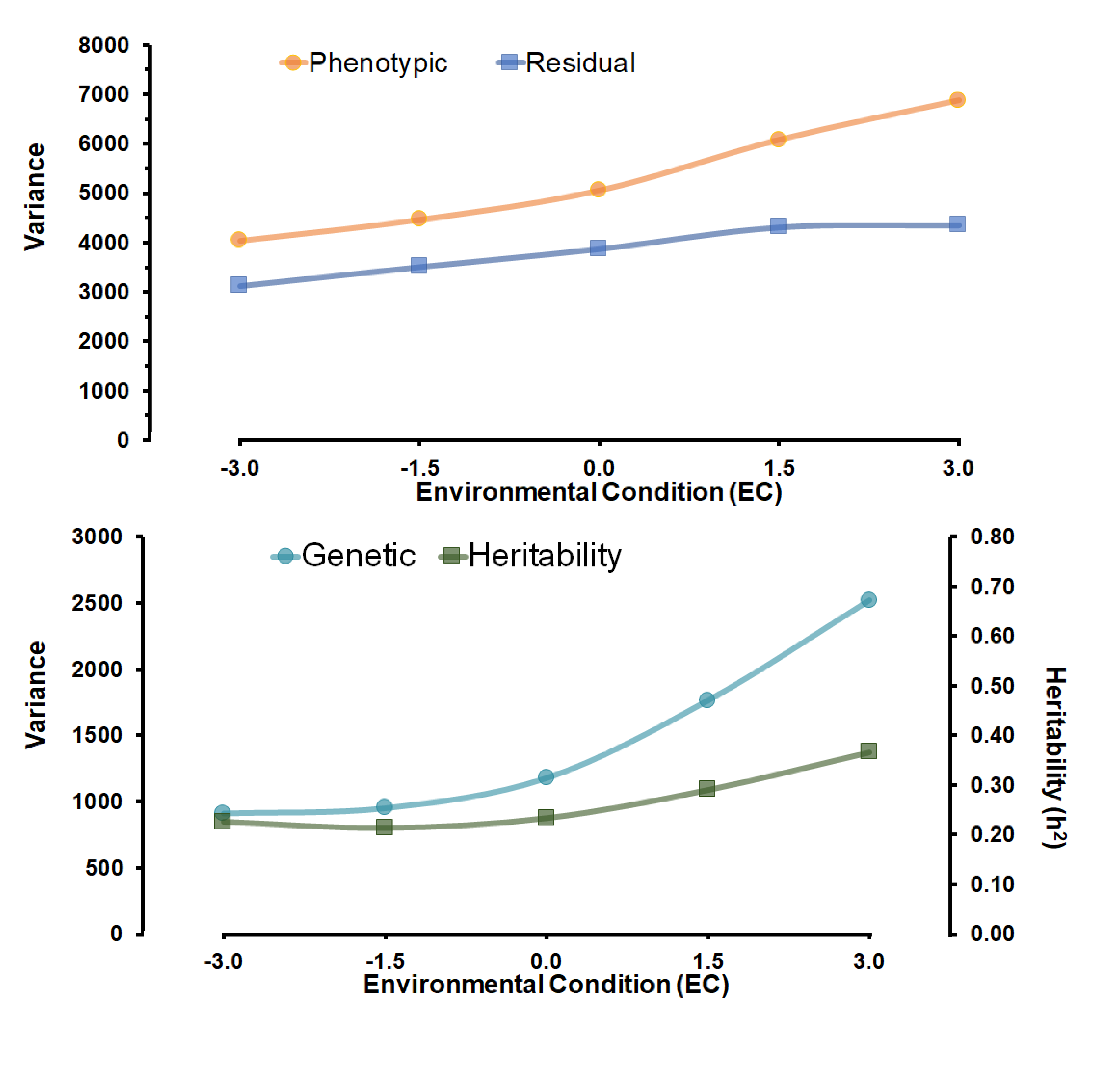


Supplementary Figure S5. Estimates of genetic parameters: A – residual and phenotypic variance and B – genetic variance and heritability for age at first calving (AFC) across Environmental condition (EC).


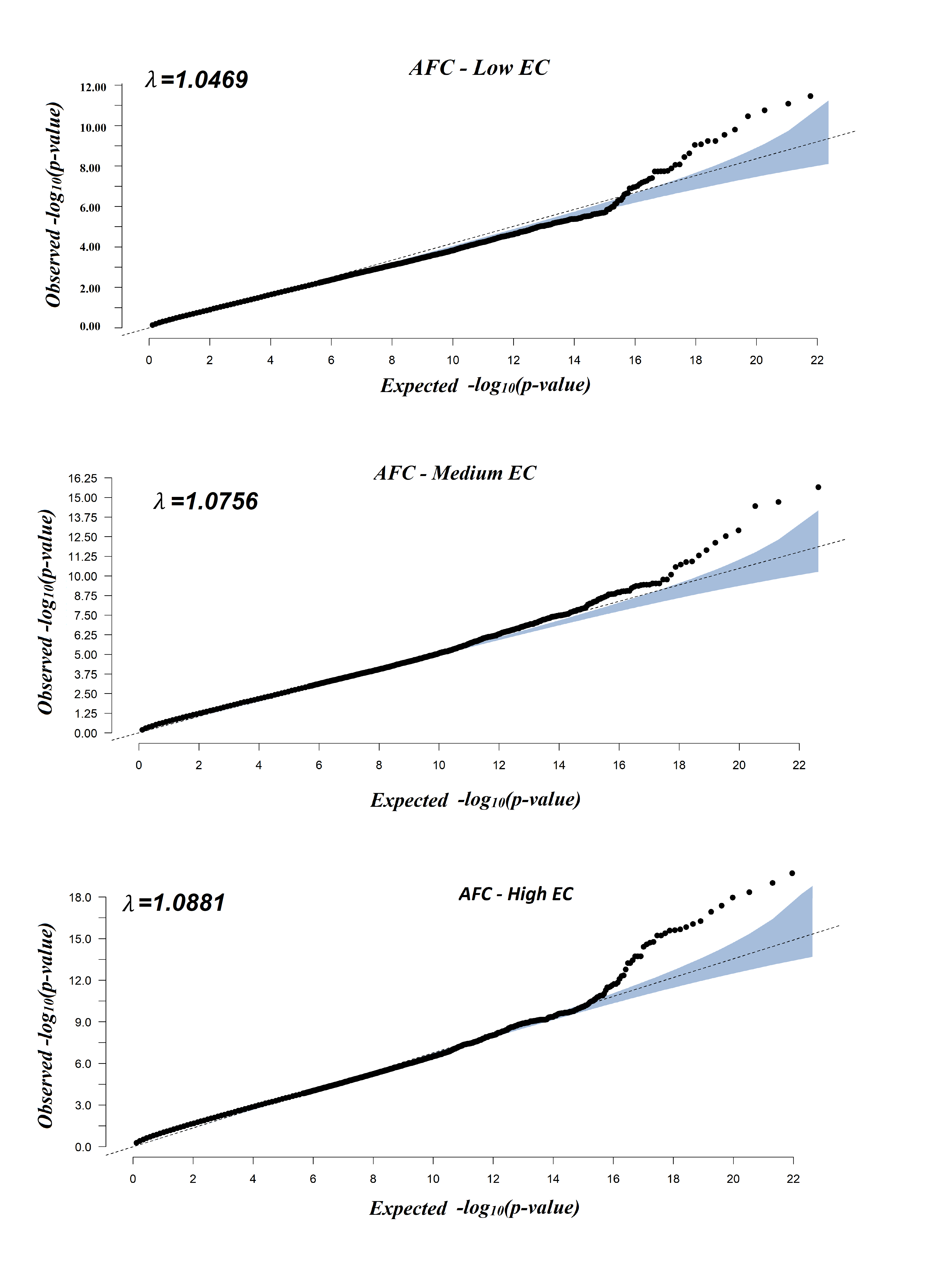


Supplementary Figure S6. The quantile–quantile (QQ) plot for the distribution of expected (under the null hypothesis) versus observed p values for SNPs. The blue area represents the 95% concentration band.

Supplementary Table S1. Genomic regions associated with age first calving (AFC) at three environmental conditions (Low, Medium and High) in Nellore cattle

| BTA | Chromosome | SNP Name | Effect | p-value | ${-log}_{10}(p-value)$ |
| --- | --- | --- | --- | --- | --- |
| *Low Environmental condition* | | | | | |
| 1 | 22921065 | BovineHD0100006753 | -0.341 | 6.97E-10 | 9.16 |
| 1 | 22923057 | BovineHD0100006756 | 0.314 | 3.18E-09 | 8.50 |
| 1 | 23032440 | BovineHD0100006789 | 0.296 | 8.34E-09 | 8.08 |
| 1 | 23021717 | BovineHD0100006784 | 0.289 | 1.22E-08 | 7.91 |
| 1 | 23038166 | BovineHD0100006791 | -0.267 | 4.11E-08 | 7.39 |
| 1 | 22863173 | BovineHD0100006741 | -0.230 | 3.29E-07 | 6.48 |
| 1 | 22868641 | BovineHD0100006742 | 0.224 | 4.43E-07 | 6.35 |
| 1 | 22943646 | Hapmap52694-rs29013683 | -0.223 | 4.82E-07 | 6.32 |
| 1 | 23024181 | BovineHD0100006785 | 0.218 | 6.07E-07 | 6.22 |
| 1 | 23030574 | BovineHD0100006788 | -0.215 | 7.40E-07 | 6.13 |
| 1 | 23020674 | BovineHD0100006783 | 0.213 | 8.42E-07 | 6.07 |
| 2 | 105357362 | BovineHD0200030238 | -0.274 | 2.84E-08 | 7.55 |
| 2 | 105048066 | BovineHD0200030233 | -0.254 | 8.54E-08 | 7.07 |
| 2 | 105044984 | BovineHD0200030235 | -0.242 | 1.63E-07 | 6.79 |
| 2 | 105051252 | BovineHD0200030236 | 0.242 | 1.69E-07 | 6.77 |
| 2 | 105360678 | BovineHD0200030239 | 0.242 | 1.69E-07 | 6.77 |
| 2 | 105048835 | BovineHD0200030234 | -0.241 | 1.73E-07 | 6.76 |
| 2 | 105061831 | BovineHD0200030240 | -0.241 | 1.73E-07 | 6.76 |
| 6 | 19490321 | BovineHD0600005405 | -0.290 | 1.16E-08 | 7.94 |
| 6 | 19494500 | BovineHD0600005407 | -0.241 | 1.73E-07 | 6.76 |
| 6 | 19539667 | BovineHD0600005425 | 0.211 | 9.22E-07 | 6.04 |
| 14 | 24941523 | BovineHD1400007242 | 0.435 | 4.02E-12 | 11.40 |
| 14 | 24980786 | BovineHD1400007251 | 0.400 | 2.75E-11 | 10.56 |
| 14 | 24989997 | BovineHD1400007252 | 0.389 | 5.14E-11 | 10.29 |
| 14 | 24922753 | BovineHD1400007237 | -0.378 | 9.44E-11 | 10.03 |
| 14 | 24821431 | BovineHD1400007208 | -0.378 | 9.44E-11 | 10.03 |
| 14 | 24897094 | BovineHD1400007228 | 0.296 | 8.41E-09 | 8.08 |
| 14 | 24855370 | BovineHD1400007216 | -0.228 | 3.63E-07 | 6.44 |
| 14 | 25069487 | BovineHD1400007272 | 0.200 | 1.72E-06 | 5.76 |
| 15 | 35344625 | BovineHD1500009720 | -0.221 | 5.18E-07 | 6.29 |
| 15 | 35642108 | BovineHD1500009837 | -0.211 | 9.01E-07 | 6.05 |
| 17 | 49836710 | BovineHD1700014162 | 0.247 | 1.26E-07 | 6.90 |
| 17 | 49833347 | BovineHD1700014160 | 0.214 | 7.96E-07 | 6.10 |
| 17 | 49647658 | BovineHD1700014099 | -0.211 | 9.00E-07 | 6.05 |
| 17 | 49707123 | BovineHD1700014115 | 0.202 | 1.48E-06 | 5.83 |
| 27 | 31972077 | Hapmap42770-BTA-103418 | 0.224 | 4.43E-07 | 6.35 |
| 27 | 31641016 | BovineHD2700008849 | -0.211 | 9.25E-07 | 6.03 |
| 27 | 31668892 | BovineHD2700008859 | 0.211 | 9.25E-07 | 6.03 |
| *Medium Environmental condition* | | | | | |
| 2 | 105357362 | BovineHD0200030238 | -0.421 | 1.13E-08 | 7.946 |
| 2 | 105042095 | BovineHD0200030231 | -0.419 | 1.41E-08 | 7.850 |
| 2 | 105061831 | BovineHD0200030240 | -0.403 | 6.85E-08 | 7.164 |
| 2 | 105039631 | BovineHD0200030227 | -0.399 | 9.62E-08 | 7.017 |
| 2 | 105048835 | BovineHD0200030234 | -0.390 | 2.28E-07 | 6.642 |
| 2 | 105367324 | BovineHD0200030242 | -0.390 | 2.46E-07 | 6.609 |
| 2 | 105044984 | BovineHD0200030235 | -0.383 | 4.84E-07 | 6.315 |
| 2 | 105041634 | BovineHD0200030230 | 0.442 | 2.71E-08 | 7.566 |
| 3 | 2121872 | BovineHD0300000579 | -0.382 | 5.07E-07 | 6.295 |
| 3 | 2119484 | BovineHD4100001783 | 0.416 | 3.46E-07 | 6.461 |
| 3 | 8343398 | BovineHD0300002744 | 0.444 | 2.26E-08 | 7.646 |
| 3 | 8402996 | BovineHD0300002770 | 0.452 | 1.09E-08 | 7.962 |
| 5 | 9470318 | BovineHD0500002692 | -0.408 | 3.92E-08 | 7.407 |
| 5 | 10764487 | BovineHD0500003108 | 0.413 | 4.42E-07 | 6.354 |
| 5 | 9461483 | BovineHD0500002689 | 0.439 | 3.71E-08 | 7.431 |
| 5 | 10867787 | BovineHD0500003172 | 0.446 | 1.82E-08 | 7.741 |
| 14 | 24941523 | BovineHD1400007242 | -0.609 | 1.33E-16 | 15.876 |
| 14 | 25069487 | BovineHD1400007272 | -0.538 | 1.29E-13 | 12.889 |
| 14 | 25075542 | BovineHD1400007273 | -0.535 | 1.80E-13 | 12.744 |
| 14 | 24911824 | BovineHD1400007233 | -0.423 | 9.95E-09 | 8.002 |
| 14 | 24897094 | BovineHD1400007228 | -0.405 | 5.40E-08 | 7.268 |
| 14 | 24900445 | BovineHD1400007229 | -0.384 | 4.19E-07 | 6.378 |
| 14 | 24855370 | BovineHD1400007216 | 0.407 | 8.59E-07 | 6.066 |
| 14 | 25079291 | BovineHD1400007274 | 0.504 | 6.86E-11 | 10.164 |
| 14 | 25026174 | BovineHD1400007262 | 0.516 | 2.18E-11 | 10.661 |
| 14 | 24821431 | BovineHD1400007208 | 0.532 | 4.23E-12 | 11.374 |
| 14 | 25050448 | BovineHD1400007266 | 0.559 | 3.33E-13 | 12.477 |
| 14 | 24922753 | BovineHD1400007237 | 0.580 | 4.23E-14 | 13.374 |
| 14 | 24906337 | BovineHD1400007231 | 0.609 | 2.43E-15 | 14.614 |
| 14 | 25036693 | BovineHD1400007265 | 0.629 | 3.51E-16 | 15.455 |
| 18 | 4794890 | BovineHD1800001417 | -0.419 | 1.47E-08 | 7.834 |
| 18 | 3863596 | BovineHD1800001134 | -0.407 | 4.58E-08 | 7.339 |
| 18 | 4892594 | BovineHD1800001462 | -0.389 | 2.59E-07 | 6.586 |
| 18 | 3847087 | BovineHD1800001131 | 0.419 | 2.61E-07 | 6.584 |
| 18 | 4810082 | BovineHD1800001430 | 0.420 | 2.38E-07 | 6.624 |
| 18 | 3824741 | BovineHD1800001123 | 0.422 | 1.97E-07 | 6.706 |
| 18 | 4637054 | BovineHD1800001443 | 0.431 | 8.33E-08 | 7.080 |
| 18 | 3025433 | BovineHD1800000851 | 0.442 | 2.80E-08 | 7.553 |
| 18 | 3026357 | BovineHD1800000852 | 0.446 | 1.93E-08 | 7.715 |
| 18 | 3023840 | BovineHD1800000850 | 0.446 | 1.93E-08 | 7.715 |
| 18 | 3080400 | BTB-01040984 | 0.460 | 4.87E-09 | 8.312 |
| *High Environmental condition* | | | | | |
| 2 | 105377464 | BovineHD0200030356 | -0.744 | 7.00E-07 | 6.155 |
| 2 | 105357362 | BovineHD0200030238 | -0.865 | 1.35E-09 | 8.870 |
| 2 | 105066298 | BovineHD0200030241 | -0.833 | 6.72E-09 | 8.172 |
| 2 | 105044984 | BovineHD0200030235 | -0.744 | 7.00E-07 | 6.155 |
| 2 | 105124680 | BovineHD0200030253 | -0.738 | 9.38E-07 | 6.028 |
| 2 | 105380413 | BovineHD0200030359 | 0.779 | 1.10E-07 | 6.960 |
| 2 | 105051252 | BovineHD0200030236 | 0.844 | 3.95E-09 | 8.404 |
| 3 | 8354360 | BovineHD0300002749 | 0.739 | 8.82E-07 | 6.055 |
| 3 | 8379666 | BovineHD0300002764 | 0.749 | 5.41E-07 | 6.267 |
| 3 | 8343398 | BovineHD0300002744 | 0.798 | 4.12E-08 | 7.385 |
| 3 | 8402996 | BovineHD0300002770 | 0.866 | 1.27E-09 | 8.895 |
| 5 | 10845026 | BovineHD0500003158 | -0.865 | 1.34E-09 | 8.873 |
| 5 | 10855850 | BovineHD0500003166 | -0.748 | 5.57E-07 | 6.254 |
| 5 | 9470318 | BovineHD0500002692 | -0.737 | 9.95E-07 | 6.002 |
| 5 | 10826367 | BovineHD0500003149 | 0.779 | 1.10E-07 | 6.960 |
| 5 | 10867787 | BovineHD0500003172 | 0.739 | 9.00E-07 | 6.046 |
| 5 | 10862100 | ARS-BFGL-NGS-117126 | 0.834 | 6.55E-09 | 8.184 |
| 5 | 10764487 | BovineHD0500003108 | 0.832 | 7.35E-09 | 8.134 |
| 14 | 24941523 | BovineHD1400007242 | -1.204 | 3.25E-17 | 16.489 |
| 14 | 25075542 | BovineHD1400007273 | -1.188 | 7.57E-17 | 16.121 |
| 14 | 25069487 | BovineHD1400007272 | -1.072 | 3.06E-14 | 13.514 |
| 14 | 23392546 | BovineHD1400006790 | -0.989 | 2.17E-12 | 11.664 |
| 14 | 24897094 | BovineHD1400007228 | -0.810 | 2.23E-08 | 7.651 |
| 14 | 24911824 | BovineHD1400007233 | -0.978 | 3.89E-12 | 11.409 |
| 14 | 24989997 | BovineHD1400007252 | -0.902 | 1.96E-10 | 9.707 |
| 14 | 24980786 | BovineHD1400007251 | -0.945 | 2.15E-11 | 10.668 |
| 14 | 24900445 | BovineHD1400007229 | -0.771 | 1.71E-07 | 6.768 |
| 14 | 24842712 | BovineHD1400024073 | -0.800 | 3.71E-08 | 7.430 |
| 14 | 25079291 | BovineHD1400007274 | 0.776 | 1.34E-07 | 6.874 |
| 14 | 24821431 | BovineHD1400007208 | 0.963 | 8.44E-12 | 11.074 |
| 14 | 24855370 | BovineHD1400007216 | 0.853 | 2.42E-09 | 8.617 |
| 14 | 24922753 | BovineHD1400007237 | 0.963 | 8.44E-12 | 11.074 |
| 14 | 25026174 | BovineHD1400007262 | 1.141 | 8.42E-16 | 15.075 |
| 14 | 25036693 | BovineHD1400007265 | 1.141 | 8.42E-16 | 15.075 |
| 14 | 24906337 | BovineHD1400007231 | 1.080 | 2.01E-14 | 13.696 |
| 14 | 25050448 | BovineHD1400007266 | 1.171 | 1.80E-16 | 15.745 |
| 18 | 4794890 | BovineHD1800001417 | -0.753 | 4.21E-07 | 6.376 |
| 18 | 3139429 | BovineHD1800000892 | -0.798 | 4.15E-08 | 7.382 |
| 18 | 3863596 | BovineHD1800001134 | -0.768 | 2.00E-07 | 6.699 |
| 18 | 3847087 | BovineHD1800001131 | 0.737 | 9.95E-07 | 6.002 |
| 18 | 4810082 | BovineHD1800001430 | 0.737 | 9.95E-07 | 6.002 |
| 18 | 3080400 | BTB-01040984 | 0.776 | 1.34E-07 | 6.874 |
| 18 | 4637054 | BovineHD1800001443 | 0.855 | 2.25E-09 | 8.648 |
| 18 | 3824741 | BovineHD1800001123 | 0.794 | 5.14E-08 | 7.289 |

Supplementary Table S2. QTLdb hits within the 100 Kb region surrounding the most significant SNP from genome-wide association in Low (-3.0 sd), Medium (0.0 sd) and High (3.0 sd) environmental condition for age at first calving (AFC) in Nellore heifer.

| Chromosome | Shared by | | | Trait | QTL_ID | QTL symbol | PUBMED  ID | Candidate Gene |
| --- | --- | --- | --- | --- | --- | --- | --- | --- |
|  | Low | Medium | High |  |  |  |  |  |
| 2 | x | X | x | Age at puberty | 21138 | PUBAGE | 22554198 | IGFBP2 |
| 2 | x | X | x | Calving to conception interval | 16220 | DAYOPEN | 21198698 | IGFBP2 |
| 2 | x | X | x | Interval from first to last insemination | 16219 | INSINT | 21198698 | IGFBP2 |
| 2 | x | X | x | Residual feed intake | 28500 | RFI | 23736061 | SMARCAL1 |
| 3 |  | X | x | Average daily gain | 35218 | ADG | 25257854 | - |
| 3 |  | X | x | Body weight (weaning) | 24711 | WWT | 24906442 | - |
| 3 |  | x | x | Calving to conception interval | 127051 | DAYOPEN | 28259397 | FCER1G |
| 3 |  | x | x | Daughter pregnancy rate | 122365 | DPR | 26923315 | FCER1G |
| 3 |  | x | x | Dry matter intake | 37430 | DMI | 25606404 | NR1I3 |
| 3 |  | x | x | First service conception | 127019 | FSC | 28259397 | FCER1G |
| 3 |  | x | x | Inseminations per conception | 127034 | CONCEPT | 28259397 | FCER1G |
| 3 |  | x | x | Longissimus muscle area | 20330 | LMA | 22394233 | - |
| 3 |  | x | x | Longissimus muscle area growth | 20433 | LMAGR | 22394233 | - |
| 3 |  | x | x | Marbling score | 20401 | MARBL | 22394233 | - |
| 3 |  | x | x | Residual feed intake | 37429 | RFI | 25606404 | NR1I3 |
| 5 |  | x | x | Calving ease | 24589 | CALEASE | 24906442 | - |
| 5 |  | x | x | Cold tolerance | 31179 | COLDT | 24362770 | - |
| 5 |  | x | x | Daughter pregnancy rate | 40769 | DPR | 21831322 | - |
| 5 |  | x | x | Length of productive life | 40771 | PL | 21831322 | - |
| 6 | x |  |  | Body weight (yearling) | 24790 | W365 | 24906442 | - |
| 14 | x | x | x | Age at puberty | 29758 | PUBAGE | 22100599 | - |
| 14 | x | x | x | Average daily gain | 20873 | ADG | 22497486 | PLAG1 |
| 14 | x | x | x | Biceps brachii weight | 125071 | BBMW | 27997562 | PLAG1 |
| 14 | x | x | x | Body weight | 20871 | BW | 22497486 | PLAG1 |
| 14 | x | x | x | Body weight (birth) | 24544 | BW | 24906442 | - |
| 14 | x | x | x | Body weight (weaning) | 24731 | WWT | 24906442 | - |
| 14 | x | x | x | Calving ease | 24578 | CALEASE | 24906442 | - |
| 14 | x | x | x | Calving ease (maternal) | 106501 | CALEASE | 27328805 | - |
| 14 | x | x | x | Carcass weight | 24631 | CWT | 24906442 | - |
| 14 | x | x | x | Growth index | 102033 | GRIND | 27136002 | - |
| 14 | x | x | x | Heifer pregnancy | 119780 | HPG | 27494397 | - |
| 14 | x | x | x | Interval to first estrus after calving | 30094 | CALEST | 22100599 | - |
| 14 | x | x | x | Intramuscular fat | 122426 | EEF | 27878829 | PLAG1 |
| 14 | x | x | x | Longissimus muscle area | 122424 | LMA | 27878829 | PLAG1 |
| 14 | x | x | x | Marbling score | 122425 | MARBL | 27878829 | PLAG1 |
| 14 | x | x | x | Residual feed intake | 56461 | RFI | 25410110 | - |
| 14 | x | x | x | Scrotal circumference | 30653 | SCRCIR | 22811567 | - |
| 14 | x | x | x | Stature | 16290 | STA | 21212230 | SDR16C6 |
| 14 | x | x | x | Subcutaneous fat | 20703 | SUBFAT | 22497247 | - |
| 14 | x | x | x | Subcutaneous rump fat thickness | 126554 | SCFR | 28118362 | - |
| 15 | x |  |  | Tridecylic acid content | 19626 | FA-C13:0 | 24156620 | PRNP |
| 17 | x |  |  | Daughter pregnancy rate | 107002 | DPR | 27209127 | - |
| 18 |  | x | x | Body capacity | 28117 | BODYCAP | 24341352 | - |
| 18 |  | x | x | Body weight gain | 68936 | BWG | 19966163 | - |
| 18 |  | x | x | Calving index | 30538 | CALVIND | 22888914 | - |
| 18 |  | x | x | Calving interval | 121658 | CALINTV | 26193888 | - |
| 18 |  | x | x | Heifer pregnancy | 119781 | HPG | 27494397 | - |
| 27 | x |  |  | Body weight (birth) | 69395 | BW | 19966163 | - |
| 27 | x |  |  | Calving ease (maternal) | 106514 | CALEASE | 27328805 | - |

Supplementary Table S3. Genes surrounding the significant genomic regions (*-log_10_(p-value) > 6*) identified in genome-wide association (GWAS) in three environmental conditions (Low, Medium and High) for age at first calving (AFC) in Nellore heifers.

| Chromosome | Position | Candidate Genes | Shared by | | | Start gene position | End gene position | Reference Snp id |
| --- | --- | --- | --- | --- | --- | --- | --- | --- |
|  |  |  | Low | Medium | High |  |  |  |
| 2 | 105044984 | SHOX | x | x | x | 104975964 | 104987392 | rs133673258 |
| 2 | 105044984 | SMARCAL1 | x | x | x | 105138334 | 105188478 | rs133673258 |
| 2 | 105357362 | IGFBP2 | x | x | x | 105338358 | 105367369 | rs719906581 |
| 2 | 105357362 | IGFBP5 | x | x | x | 105378991 | 105397646 | rs719906582 |
| 3 | 2119484 | ILDR2 |  | x |  | 1955064 | 2022631 | rs525380276 |
| 3 | 2119484 | TADA1 |  | x |  | 2089527 | 2105290 | rs478735272 |
| 3 | 2121872 | POGK |  | x |  | 2107192 | 2126252 | rs378083198 |
| 3 | 8343398 | ADAMTS4 |  | x | x | 8322938 | 8331146 | rs136680939 |
| 3 | 8343398 | APOA2 |  | x | x | 8301476 | 8302729 | rs136680939 |
| 3 | 8343398 | ARHGAP30 |  | x | x | 8439001 | 8453973 | rs136680939 |
| 3 | 8343398 | B4GALT3 |  | x | x | 8340547 | 8345532 | rs136680939 |
| 3 | 8343398 | DEDD |  | x | x | 8381806 | 8392000 | rs136680939 |
| 3 | 8343398 | FCER1G |  | x | x | 8305544 | 8308776 | rs136680939 |
| 3 | 8343398 | KLHDC9 |  | x | x | 8407875 | 8409695 | rs136680939 |
| 3 | 8343398 | NDUFS2 |  | x | x | 8309350 | 8318876 | rs136680939 |
| 3 | 8343398 | NECTIN4 |  | x | x | 8421828 | 8438222 | rs136680939 |
| 3 | 8343398 | NIT1 |  | x | x | 8392622 | 8395532 | rs136680939 |
| 3 | 8343398 | NR1I3 |  | x | x | 8290360 | 8295271 | rs136680939 |
| 3 | 8343398 | PCP4L1 |  | x | x | 8249981 | 8276724 | rs136680939 |
| 3 | 8343398 | PFDN2 |  | x | x | 8395605 | 8407551 | rs136680939 |
| 3 | 8343398 | PPOX |  | x | x | 8345630 | 8349334 | rs136680939 |
| 3 | 8343398 | TOMM40L |  | x | x | 8294406 | 8298900 | rs136680939 |
| 3 | 8343398 | UFC1 |  | x | x | 8356956 | 8360652 | rs136680939 |
| 3 | 8343398 | USP21 |  | x | x | 8350180 | 8356345 | rs136680939 |
| 3 | 8356909 | USF1 |  | x | x | 8455700 | 8461692 | rs132836368 |
| 3 | 8379666 | TSTD1 |  | x | x | 8461937 | 8463395 | rs110841380 |
| 3 | 8402996 | F11R |  | x | x | 8483556 | 8508122 | rs134229906 |
| 5 | 9461483 | PPP1R12A |  | x |  | 9347411 | 9511533 | rs43428515 |
| 5 | 10862100 | ACSS3 |  | x | x | 10731241 | 10905490 | rs110828656 |
| 6 | 19490321 | DKK2 | x |  |  | 19381413 | 19511148 | rs42778919 |
| 14 | 23392546 | OPRK1 |  |  | x | 23373836 | 23395443 | rs135612754 |
| 14 | 24821431 | LYN | x | x | x | 24847257 | 24920713 | rs136350664 |
| 14 | 24821431 | TGS1 | x | x | x | 24747219 | 24772713 | rs136350664 |
| 14 | 24842712 | TMEM68 |  |  | x | 24711327 | 24747118 | rs137365107 |
| 14 | 24855370 | RPS20 | x | x | x | 24955079 | 24956324 | rs137783321 |
| 14 | 24897094 | MOS | x | x | x | 24975950 | 24976948 | rs135626029 |
| 14 | 24911824 | PLAG1 | x | x | x | 25007291 | 25009296 | rs137044774 |
| 14 | 24980786 | CHCHD7 | x | x | x | 25052885 | 25058779 | rs135318045 |
| 14 | 25026174 | SDR16C5 |  | x | x | 25105062 | 25117554 | rs136828442 |
| 14 | 25069487 | SDR16C6 | x | x | x | 25153583 | 25179651 | rs135114806 |
| 15 | 35344625 | KCNC1 | x |  |  | 35272154 | 35317245 | rs109389621 |
| 15 | 35344625 | MYOD1 | x |  |  | 35331401 | 35334046 | rs109389621 |
| 15 | 35344625 | SERGEF | x |  |  | 35019670 | 35267343 | rs109389621 |
| 15 | 35642108 | ABCC8 | x |  |  | 35567687 | 35646400 | rs110915536 |
| 15 | 35642108 | KCNJ11 | x |  |  | 35650715 | 35653362 | rs110915536 |
| 15 | 35642108 | NUCB2 | x |  |  | 35693278 | 35715902 | rs110915536 |
| 15 | 35642108 | USH1C | x |  |  | 35500365 | 35548981 | rs110915536 |
| 18 | 3023840 | ADAT1 |  | x | x | 2944867 | 2960921 | rs722724047 |
| 18 | 3025433 | KARS |  | x | x | 2962722 | 2976537 | rs207905155 |
| 18 | 3026357 | TERF2IP |  | x | x | 2976672 | 2983194 | rs876058375 |
| 18 | 3824741 | CNTNAP4 |  | x | x | 3505270 | 3744371 | rs110783254 |
| 18 | 4637054 | ADAMTS18 |  | x | x | 4522898 | 4666475 | rs133972679 |
| 18 | 4892594 | NUDT7 |  | x | x | 4990463 | 5003016 | rs110255213 |
| 27 | 31972077 | KCNU1 | x |  |  | 31849127 | 31993694 | rs41615011 |

Supplementary Table S4. Gene ontology enrichment analysis for biological processes of the genes identified for age at first calving (AFC) in Low environmental condition.

| ID | Description | pvalue | qvalue | geneID |
| --- | --- | --- | --- | --- |
| GO:0060251 | regulation of glial cell proliferation | 0.0003 | 0.0106 | LYN, PLAG1, ABCC8 |
| GO:2001259 | positive regulation of cation channel activity | 0.0006 | 0.0143 | KCNC1, KCNJ11, ABCC8 |
| GO:0010817 | regulation of hormone levels | 0.0010 | 0.0175 | SDR16C5, KCNJ11, LYN |
| GO:0071805 | potassium ion transmembrane transport | 0.0015 | 0.0212 | KCNC1, KCNJ11, ABCC8, KCNU1 |
| GO:0014888 | striated muscle adaptation | 0.0015 | 0.0212 | IGFBP5, MYOD1 |
| GO:0043627 | response to estrogen | 0.0019 | 0.0235 | KCNJ11, MYOD1 |
| GO:1903818 | positive regulation of voltage-gated potassium channel activity | 0.0021 | 0.0243 | KCNC1, ABCC8 |
| GO:0014013 | regulation of gliogenesis | 0.0038 | 0.0294 | LYN, PLAG1, ABCC8 |
| GO:1901018 | positive regulation of potassium ion transmembrane transporter activity | 0.0055 | 0.0355 | KCNC1, ABCC8 |
| GO:0050709 | negative regulation of protein secretion | 0.0048 | 0.0326 | KCNJ11, ABCC8, SERGEF |
| GO:0043501 | skeletal muscle adaptation | 0.0060 | 0.0384 | IGFBP5, MYOD1 |
| GO:1904062 | regulation of cation transmembrane transport | 0.0060 | 0.0385 | KCNC1, KCNJ11, LYN, ABCC8 |
| GO:1904064 | positive regulation of cation transmembrane transport | 0.0062 | 0.0397 | KCNC1, KCNJ11, ABCC8 |
| GO:0010817 | response to estradiol | 0.0063 | 0.0408 | IGFBP2, KCNJ11, MYOD1 |
| GO:0002792 | negative regulation of peptide secretion | 0.0064 | 0.0420 | KCNJ11, ABCC8, SERGEF |
| GO:0046888 | negative regulation of hormone secretion | 0.0057 | 0.0368 | KCNJ11, ABCC8 |
| GO:0007565 | female pregnancy | 0.0075 | 0.0429 | IGFBP2, IGFBP5, ABCC8 |
| GO:0033500 | carbohydrate homeostasis | 0.0055 | 0.0354 | IGFBP5, KCNJ11, ABCC8 |
| GO:0090278 | negative regulation of peptide hormone secretion | 0.0046 | 0.0317 | KCNJ11, ABCC8 |
| GO:0046676 | negative regulation of insulin secretion | 0.0055 | 0.0358 | KCNJ11, ABCC8 |
| GO:0042593 | glucose homeostasis | 0.0050 | 0.0331 | IGFBP5, KCNJ11, ABCC8 |
| GO:0043567 | regulation of insulin-like growth factor receptor signaling pathway | 0.0030 | 0.0274 | IGFBP2, IGFBP5 |
| GO:0048009 | insulin-like growth factor receptor signaling pathway | 0.0029 | 0.0272 | IGFBP2, IGFBP5 |

Supplementary Table S5. Gene ontology enrichment analysis for biological processes of the genes identified for age at first calving (AFC) in Medium environmental condition.

| ID | Description | pvalue | qvalue | geneID |
| --- | --- | --- | --- | --- |
| GO:0071396 | cellular response to lipid | 0.00748 | 0.012856 | LYN, NR1I3 |
| GO:0031669 | cellular response to nutrient levels | 0.00616 | 0.010954 | SNTG1, USF1 |
| GO:0060986 | endocrine hormone secretion | 0.00712 | 0.012335 | IGFBP2, IGFBP5 |
| GO:0044849 | estrous cycle | 0.01178 | 0.019058 | IGFBP2, IGFBP5 |
| GO:0007565 | female pregnancy | 0.00976 | 0.016149 | IGFBP2, IGFBP5 |
| GO:0014009 | glial cell proliferation | 0.00017 | 0.002327 | LYN, PLAG1 |
| GO:0048009 | insulin-like growth factor receptor signaling pathway | 0.01167 | 0.018910 | IGFBP2, IGFBP5 |
| GO:0044706 | multi-multicellular organism process | 0.02516 | 0.038333 | IGFBP2, IGFBP5 |
| GO:0042698 | ovulation cycle | 0.00024 | 0.002428 | IGFBP2, IGFBP5 |
| GO:0014015 | positive regulation of gliogenesis | 0.01559 | 0.024552 | LYN, PLAG1 |
| GO:0050878 | regulation of body fluid levels | 0.00189 | 0.004799 | ADAMTS18, FCER1G, LYN, F11R, USF1 |
| GO:0032276 | regulation of gonadotropin secretion | 0.00421 | 0.00814 | IGFBP2, IGFBP5 |
| GO:0043567 | regulation of insulin-like growth factor receptor signaling pathway | 0.01292 | 0.020696 | IGFBP2, IGFBP5 |
| GO:0043627 | response to estrogen | 0.0283 | 0.042861 | PLAG1, DEDD |
| GO:0060416 | response to growth hormone | 0.02319 | 0.035494 | LYN, IGFBP2, IGFBP5 |
| GO:0032868 | response to insulin | 0.02553 | 0.038873 | LYN, USF1 |
| GO:0043434 | response to peptide hormone | 0.0011 | 0.00366 | LYN, USF1 |
| GO:0043501 | skeletal muscle adaptation | 0.00477 | 0.008958 | IGFBP5, IGFBP2 |
| GO:0022414 | reproductive process | 0.0017 | 0.004528 | PLAG1, DEDD |
| GO:0000187 | activation of MAPK activity | 0.01309 | 0.020942 | KARS, MOS |
| GO:0006950 | response to stress | 0.00756 | 0.01298 | ADAMTS18, FCER1G, LYN, NDUFS2, F11R, UFC1, TERF2IP, USF1 |
| GO:0042063 | gliogenesis | 0.01103 | 0.017981 | LYN, PLAG1 |

Supplementary Table S6. Gene ontology enrichment analysis for biological processes of the genes identified for age at first calving (AFC) in High environmental condition.

| ID | Description | pvalue | qvalue | geneID |
| --- | --- | --- | --- | --- |
| GO:0044242 | cellular lipid catabolic process | 0.0076 | 0.0376 | APOA2, NUDT7 |
| GO:0071378 | cellular response to growth hormone stimulus | 0.0053 | 0.0359 | LYN, PLAG1 |
| GO:0031670 | cellular response to nutrient | 0.0093 | 0.0388 | USF1, IGFBP5 |
| GO:0048878 | chemical homeostasis | 0.0085 | 0.0382 | IGFBP5, USF1, APOA2 |
| GO:0042632 | cholesterol homeostasis | 0.0011 | 0.0328 | APOA2, NR1I3 |
| GO:0007565 | female pregnancy | 0.0063 | 0.0366 | IGFBP2, IGFBP5 |
| GO:0042593 | glucose homeostasis | 0.0082 | 0.038 | IGFBP5, USF1 |
| GO:0006006 | glucose metabolic process | 0.0064 | 0.0367 | IGFBP5, USF1 |
| GO:0032274 | gonadotropin secretion | 0.0219 | 0.0481 | OPRK1 |
| GO:0007599 | hemostasis | 0.0038 | 0.0348 | FCER1G, LYN, USF1 |
| GO:0009914 | hormone transport | 0.0155 | 0.0433 | LYN, OPRK1 |
| GO:0048009 | insulin-like growth factor receptor signaling pathway | 0.0003 | 0.0322 | IGFBP2, IGFBP5 |
| GO:0016042 | lipid catabolic process | 0.0156 | 0.0434 | APOA2, NUDT7 |
| GO:0055088 | lipid homeostasis | 0.0002 | 0.0321 | APOA2, USF1, NR1I3 |
| GO:0032275 | luteinizing hormone secretion | 0.0122 | 0.0409 | OPRK1 |
| GO:0060192 | negative regulation of lipase activity | 0.0236 | 0.0492 | APOA2 |
| GO:0046461 | neutral lipid catabolic process | 0.0087 | 0.0384 | NDUFS2, APOA2, PPOX, SDR16C6 |
| GO:0042698 | ovulation cycle | 0.0136 | 0.0419 | OPRK1, IGFBP5 |
| GO:0045834 | positive regulation of lipid metabolic process | 0.0033 | 0.0342 | APOA2, LYN |
| GO:0032276 | regulation of gonadotropin secretion | 0.0125 | 0.0411 | OPRK1, IGFBP2, IGFBP5 |
| GO:0040008 | regulation of growth | 0.0146 | 0.0427 | IGFBP2, IGFBP5 |
| GO:0043567 | regulation of insulin-like growth factor receptor signaling pathway | 0.0001 | 0.0321 | IGFBP2, IGFBP5 |
| GO:0019216 | regulation of lipid metabolic process | 0.0044 | 0.0352 | APOA2, LYN, TGS1 |
| GO:1903305 | regulation of regulated secretory pathway | 0.0029 | 0.0341 | FCER1G, LYN |
| GO:0051046 | regulation of secretion | 0.0035 | 0.0346 | FCER1G, APOA2, OPRK1 |
| GO:0031960 | response to corticosteroid | 0.0048 | 0.0355 | APOA2, IGFBP2 |
| GO:0043627 | response to estrogen | 0.0031 | 0.0321 | APOA2, IGFBP2, OPRK1 |
| GO:0060416 | response to growth hormone | 0.0034 | 0.0322 | IGFBP5, LYN |
| GO:0032868 | response to insulin | 0.0017 | 0.0332 | LYN, OPRK1, USF1 |
| GO:0043434 | response to peptide hormone | 0.0013 | 0.033 | IGFBP5, LYN, OPRK1, USF1 |
| GO:0048545 | response to steroid hormone | 0.0052 | 0.0358 | APOA2, IGFBP2, NR1I3 |
| GO:0055092 | sterol homeostasis | 0.0011 | 0.0328 | APOA2, NR1I3 |
| GO:0022414 | reproductive process | 0.0031 | 0.0343 | OPRK1, PLAG1, DEDD |
| GO:0042063 | gliogenesis | 0.0164 | 0.0440 | LYN, PLAG1 |
| GO:0010817 | regulation of hormone levels | 0.0167 | 0.0442 | SDR16C5, LYN, OPRK1 |
| GO:0009719 | response to endogenous stimulus | 0.0215 | 0.0477 | LYN, OPRK1, F11R, USF1, NR1I3 |
